# Clinical and genetic features of multiple primary tumours cohorts with a renal cell carcinoma: Implications for molecular genetic investigations

**DOI:** 10.1002/ijc.70085

**Published:** 2025-08-22

**Authors:** Huairen Zhang, Bryndis Yngvadottir, Avgi Andreou, Yasemin Cole, Emma R. Woodward, James Whitworth, Eamonn R. Maher

**Affiliations:** ^1^ Department of Medical Genetics, School of Clinical Medicine University of Cambridge Cambridge UK; ^2^ Manchester Centre for Genomic Medicine, and Division of Evolution, Infection and Genomics University of Manchester Manchester UK; ^3^ Aston Medical School, College of Health and Life Sciences Aston University Birmingham UK

**Keywords:** cancer, genetic testing, genomics, kidney, mutation

## Abstract

Multiple primary tumours (MPT) is a risk factor for an underlying predisposition to cancer. Renal cell carcinoma (RCC) occurs in several hereditary cancer disorder syndromes, and RCC‐related MPT comprise individuals with multiple primary renal tumours (MPRT) and those with RCC plus a non‐renal tumour (MPT:RCC + X). Excluding rare syndromic causes, knowledge of the genetic architecture of MPRT/MPT:RCC + X is limited. To inform diagnostic approaches to MPRT/MPT:RCC + X, we present the findings of comprehensive genomic analysis in 534 individuals. The presence/absence of variants in cancer susceptibility genes (CSGs) from exome/genome sequencing was then correlated with data on age, sex, tumour types, and RCC histopathology in 93 participants with MPRT and 441 with MPT:RCC + X. 7.5% of participants with MPRT and 6.1% in participants with MPT:RCC + X had germline CSG pathogenic variants, and the diagnostic yields increased to 9.4% and 10.4%, respectively, in cases with RCC <66 years. Excluding participants with environmental carcinogen‐linked cancers increased the diagnostic yield in MPT:RCC + X to 12.9%. Pathogenic variants were mostly in CSGs known to predispose to RCC, but in almost half of these cases, typical extrarenal tumours were not present. In conclusion, our findings support amended eligibility criteria for diagnostic testing in MPRT and wider eligibility criteria for testing in MPT:RCC + X, with RCC <66 years.

## What's New?

Current knowledge of the genetic architecture underlying the development of renal cell carcinoma‐related multiple primary tumours is limited. The authors comprehensively analysed the results of exome/genome sequencing cohorts including 534 patients to better define genotype–phenotype correlations. A germline pathogenic variant in a cancer susceptibility gene was observed in 9.4% of the patients with a renal cell carcinoma diagnosed before the age of 66 with other primary renal tumours and 10.4% of those who also developed another primary tumour type. The authors suggest new eligibility criteria for genetic testing in patients with multiple primary tumours including a renal cell carcinoma.

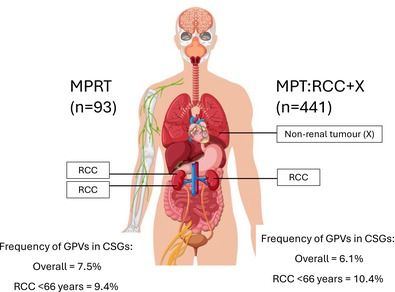

**Abbreviations**
ACMG: American College of Medical Genetics and Genomics classification
adCSG: cancer susceptibility genes inherited in autosomal dominant pattern
AF: allele frequency
AUC: area under the curve
CADD: combined annotation dependent depletion
CNV: copy number variant
CRG: cancer related gene
CSG: cancer susceptibility gene
ES: exome sequencing
gnomAD: genome aggregation database
GS: genome sequencing
HLRCC: hereditary leiomyomatosis and renal cell cancer syndromes
HPRC: hereditary papillary renal cancer
LP: likely pathogenic
MPRT: multiple primary renal tumours
MPT: multiple primary tumours
MPT‐RCC+X: multiple primary tumours including a renal cell carcinoma and non‐renal tumour
OMIM: online Mendelian inheritance in man database
P: pathogenic
pLoF: predicted loss of function
PolyPhen: polymorphism phenotyping
RCC: renal cell carcinoma
SDH: succinate dehydrogenase complex
SIFT: sorting intolerant for tolerant
SNP: single nucleotide polymorphism
TSC: tuberous sclerosis complex
VEP: variant effect predictor
VHL: Von Hippel–Lindau disease
VUS: variant with uncertain significance
100kGP: 100,000 Genomes Project in UK

## 1 INTRODUCTION

Multiple primary tumours (MPT) are an increasing issue amongst cancer patients and survivors with a reported general prevalence of 2%–17%, though this varies by primary cancer site and length of follow‐up.^1^, ^2^, ^3^, ^4^ The rising incidence of MPT has been attributed to multiple factors including better cancer survival rates, improved diagnostic techniques, and surveillance and pro‐oncogenic effects of radiotherapy/chemotherapy.^1^, ^3^ MPT is also a well‐recognised feature of many inherited cancer predisposition disorders.^5^ Globally, around 400,000 cases of kidney cancer occur each year, with renal cell carcinoma (RCC) being the most common form.^6^ In patients with RCC as part of a MPT phenotype, two major subgroups can be delineated: first, those with two or more primary RCC (synchronous or metachronous, with no extrarenal tumour; herein referred to as MPRT) and, second, individuals with RCC and a second extra‐renal tumour (herein referred to as MPT:RCC + X). Over recent decades, the incidence of RCC has been increasing, in part through incidental diagnoses during abdominal imaging, and numbers of individuals with MPRT and with MPT:RCC + X have also risen.^7^ In a recent systematic literature review of MPRT, we found that, compared to unselected cases of RCC, those with MPRT are more likely male, to have an early age at diagnosis (<46 years) and have the histopathologic papillary subtype of RCC.^8^ Both a younger age at cancer diagnosis and the occurrence of MPT are features of hereditary RCC predisposition syndromes, and ~15% of patients with MPRT reported in the literature had a family history of cancer or had been diagnosed with a hereditary RCC‐associated syndrome.^8^

The most common genetic causes of MPRT reported in the literature‐based series were the better‐known hereditary RCC cancer syndromes such as von Hippel–Lindau (VHL) disease, Birt‐Hogg‐Dube syndrome, hereditary papillary renal cancer (HPRC) with *MET* variants, tuberous sclerosis complex (TSC) and hereditary leiomyomatosis and renal cell cancer syndrome (HLRCC). With the exception of HPRC, the diagnosis of these hereditary RCC syndromes is often indicated by the presence and type of extra‐renal tumours and non‐neoplastic clinical features.^8^ However, penetrance is often incomplete in inherited RCC syndromes, and the presence of syndromic features can be overlooked, leading to the underdiagnosis of underlying genetic causes of MPRT or MPT:RCC + X.^8^ Large‐scale genomic sequencing studies of unselected patients with RCC have shown that the frequency of germline pathogenic variants in cancer susceptibility genes (CSG) can be substantially higher than the number of cases diagnosed because of clinical features of a specific hereditary cancer syndrome.^9^ Previously, genome sequencing (GS) in a series of individuals with MPT of many different types and negative clinical diagnostic genetic testing identified likely clinically relevant candidate pathogenic/likely pathogenic (P/LP) germline variants in CSG in ~15% of participants.^5^ Therefore, it can be hypothesised that comprehensive genomic analysis of MPRT and MPT:RCC + X cohorts might reveal clinically unsuspected germline P/LP variants in CSGs that would impact patient management and cancer risks in their family. However, in a recent literature review of MPRT, we did not identify large‐scale comprehensive genomic studies to define the genetic architecture of MPRT.^8^ Therefore, we proceeded to review the results of exome/GS cohorts of patients (*n* = 534) with MPRT and MPT:RCC + X from the 100,000 Genomes Project (100kGP) and a local study, human genetic disease (HGDis‐RCC). Whilst the 100kGP study recruited participants with cancer or rare disorders, the HGDis‐RCC study recruited participants with features that might suggest an underlying genetic cause. The principal aim of this study was to define genotype–phenotype correlations for MPRT and MPT:RCC + X and identify criteria that might be used to guide the application of genetic testing in these conditions.

## 2 METHODS

### 2.1 Participants

A total of 534 participants, with MPRT or MPT:RCC + X, were ascertained from three genomically characterised study cohorts. First, a cohort of patients (29 with MPRT and 40 with MPT:RCC + X) who underwent GS or exome sequencing (ES) as reported previously.^5^, ^10^ A second group of patients (42 with MPRT and 242 with MPT:RCC + X) with GS was ascertained from the UK 100,000 Genomes Project (100kGP) that we had reported previously.^11^ Third, 181 individuals with MPRT (*n* = 22) or MPT:RCC + X (*n* = 159) with GS or ES from in‐house or 100kGP studies that were not reported previously. MPRT was defined as the occurrence of two or more primary renal tumours without evidence of intrarenal metastases. A total of 17 participants from the 100kGP cohort with both MPRT and an extra‐renal tumour were allocated to the MPRT cohort. In total, the MPRT cohort consisted of 93 participants analysed by ES (*n* = 17) or GS (*n* = 76) and the MPT:RCC + X cohort consisted of 441 participants (ES = 3 and GS = 438).

The version 18 (21‐12‐2023) of 100kGP data was used to identify patients with MPT based on their recorded cancer site (e.g., renal) and cancer type (primary). Additionally, since participants in 100kGP may have had multiple samplings of the same tumour, tumour ID was checked to confirm MPRT. Only participants with MPRT or MPT:RCC + X for whom GS or ES data were available were included in this study.

### 2.2 Molecular and bioinformatic analysis

#### 2.2.1 Sequencing

##### Variant collection, annotation, and filtering

Details of exome/sequencing procedures are described in Supplementary Methods, and the sequencing coverage and quality statistics for each sample are summarised in Supplementary Table 1. Germline variants in 550 cancer‐related genes (CRG) were analysed (Supplementary Table 2). The CRG list was compiled from gene lists of CRGs analysed for somatic variants in cancer gene panels^10^, ^12^, ^13^ with a focus on genes mutated in RCC.^14^, ^15^ The CRG list contained 105 CSGs and potential CSGs (Supplementary Table 2). Results for 30 participants with MPRT tested for germline variants in 118 non‐RCC CSG^10^ and 52 (MPRT = 12 and MPT:RCC + X = 40) participants recruited to the HGD‐RCC for germline variants in 83 CSGs were reported previously^5^ and all participants (MPRT = 57 and MPT:RCC + X = 389) from the 100kGP cohort were included in a report of germline variants in 121 CSGs.^9^

The germline SNP/indel variants in the CRG were accessed from the VCF file of the participants using BCF tools, and only those with FILTER = PASS and quality score ≥30 were included (as described previously).^9^ CNVs that overlapped CRGs were called by both CANVAS and MANTA from the BAM file of the participants, and only those with quality >10 by CANVAS and supporting reads (sum of spanning paired‐reads and the split reads for both reference allele and alternative allele) ≥15 by MANTA were included.^16^, ^17^ To increase CNV calling specificity, only consensus deletions called by both CANVAS and MANTA with length >50 bp were included for variant annotation and filtering.^9^ This strategy differed from that on the same participants that were included in a previous study which applied a three‐caller (Delly, MANTA, and Lumpy) pipeline.^9^ An SV filter of supporting reads ≥15 by MANTA was used in both studies, but in the current study, a quality filter of >10 by CANVAS was additionally applied.

#### 2.2.2 Variant annotation and filtering

The germline SNP/indel variants were annotated by variant effect predictor (VEP).^18^ Only the variants in the protein coding region of CRG and with allele frequency (AF) in genome aggregation database (gnomAD)^19^ ≤ 0.005, AF in Hardy–Weinberg equilibrium ≥ 0.001, sorting intolerant from tolerant (SIFT)^20^ = deleterious, polymorphism phenotyping (PolyPhen)^21^ = probably damaging, and combined annotation dependent depletion (CADD)^22^ ≥ 20, or the SNP/indel variants recorded as pathogenic or likely pathogenic (P/LP) in ClinVar^23^ were further studied. This stringency filter for pathogenicity is also the same as the one used previously.^9^

The germline CNV variants were annotated with AnnotSV.^24^ Only the CNV containing the exonic region of the CRG and with AF <0.05 and the American College of Medical Genetics and Genomics (ACMG) classification^25^ of P/LP remained. Such filters for pathogenicity are also used in the previous study,^9^ but additional filters for deletion variants, including predicted loss of function (pLoF) >0.9 and gene intolerance (*Z* score) >0, were applied. Moreover, the DECIPHER database was referred to for further assessment of the clinical significance of a CNV.^26^

#### 2.2.3 Variant analysis

The evaluation of pathogenicity of the previously unreviewed variants was performed based on the ACMG/AMP standard and guidelines and utilising ClinVar, Cosmic, InterVar, online Mendelian inheritance in man database (OMIM), gnomAD, and other genomic resources.^19^, ^23^, ^27^, ^28^, ^29^ For stop‐gain and frameshift indel variants, if the truncating variant was on the last exon of the gene, its loss‐of‐function (pLoF) was predicted using the LoFtool and LOFTEE.^30^, ^31^

### 2.3 Statistical analysis

Fishers exact test (for sample size <10) or chi‐square test (for sample size ≥10) were used to compare the genetic yields (the percentage of participants with P/LP germline variants in CSG) between participants with MPRT and participants with MPT:RCC + X, as well as between male and female participants. ANOVA test was used to compare the age distribution of general participants with RCC, participants with MPRT, and participants with MPT:RCC + X of different gender. Linear regression analysis was utilised to evaluate the association between the genetic yield and age, gender, and RCC histopathology. Area under the curve (AUC) analysis was used to evaluate clinical criteria that might be used to target genetic testing to subgroups of individuals with MPRT and MPT:RCC + X.

## 3 RESULTS

### 3.1 Clinical characteristic of study participants

**FIGURE 1:**
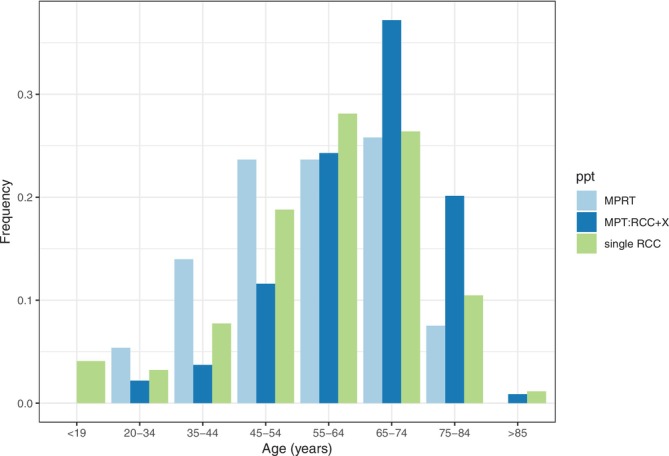
The age distribution of age at diagnosis of the (first) renal cell carcinoma (RCC) in patients with multiple primary renal tumours (MPRT) and patients with RCC and an extra renal tumour (MPT:RCC + X) compared with the patients with RCC without MPRT or MPT:RCC + X (“single RCC” in 100kGP).^2^ The mean age at diagnosis of RCC in the general participants in 100kGP is 57.9 years of which the mean diagnostic age for males is 58.3 and 57.2 years for females.

A total of 534 participants with MPRT (*n* = 93, mean age of the first RCC 59.3 years and second RCC 60.3 years; 67 males and 26 females; mean ages of first RCC 56.5, 55.4 years and second RCC 58.7, 64.5, respectively) or MPT:RCC + X (*n* = 441, mean age 62.5 years; 268 males and 173 females; mean ages 65.0, 66.6 years, respectively) were studied. The mean age of diagnosis of the first RCC was younger in participants with MPRT than in those with MPT:RCC + X (56.5 vs. 65.0 years, *p* = 4.6 × 10^−6^) (Figure 1). Comparisons of mean age at RCC (57.9 years) in 100kGP participants without MPRT or MPT:RCC + X showed no significant difference (*p* > .05) to the MPRT cohort but a significantly earlier age at onset than in the MPT:RCC + X cohort (*p* = 3.1 × 10^−11^).

**FIGURE 2:**
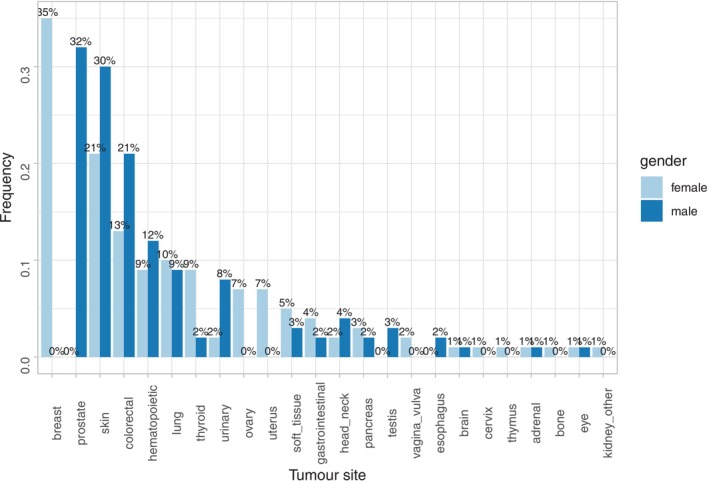
The distribution of the extra renal neoplasm in male and female participants with a renal and an extra renal neoplasm (MPT:RCC + X).

Most (62%, 49/79) participants with MPRT had presented with synchronous MPRT, and in those with metachronous MPRT, the second RCC was, on average, diagnosed 6.4 years (range: 1–24 years) after the first RCC. In participants with MPT:RCC + X, the most common (present in ≥10% of participants) extra‐renal tumours observed in females were breast (35%), skin (20%), colorectal (13%), and lung (10%); and in males, prostate (30%), skin (28%), colorectal (21%), and haematopoietic neoplasia (12%) (Figure 2). Almost a quarter, 23% (100/441; 39 females and 61 males), of participants with MPT‐RCC + X had more than one extra‐renal tumour (e.g., breast + colorectal cancers (*n* = 4), breast + skin cancers (*n* = 4) and thyroid + uterine cancers (*n* = 4) in females and prostate + skin cancer (*n* = 15) in males). The mean age at diagnosis of the most common extra‐renal neoplasms in 100kGP participants with MPT:RCC + X was not significantly younger than the mean age of all participants with the relevant cancer recruited to the cancer arm of the 100kGP (e.g., 56.4 and 56.8 years, respectively, for breast cancer [Supplementary Table 3]).

RCC histopathology results were available for 433 participants (excluding those with not otherwise specified histology [NOS]). For the MPT:RCC + X, 76% (270/355) of RCC were classified as clear cell RCC, 13% papillary RCC, and 7% chromophobe RCC.^32^, ^33^ For participants with MPRT (assuming clear cell RCC accounts for ≥70% of RCC and papillary ~15%), 37% (29/78) had multiple clear cell RCC (expected by chance >49%), 10% (8/78) multiple papillary RCC (expected by chance ~2%) and 3% (2/78) multiple chromophobe RCC, with the remainder (37%) having combinations of different renal histopathologies. As for the RCC laterality in participants with MPRT, 87% (48/55) participants had bilateral MPRT and 13% (7/55) unilateral MPRT. For those with bilateral MPRT, 33% (16/48) had multiple clear cell RCC (expected by chance >49%), 10% (5/48) multiple papillary RCC (expected by chance ~2%) and 2% (1/48) multiple chromophobe RCC, with the remainder (42%) having combinations of different renal histopathologies.

### 3.2 Germline variants in cancer related genes in individuals with MPRT

After the application of a series of stringent filters, 122 variants in 101 of 550 CRG were prioritised. These were divided into two groups according to whether the relevant genes were confirmed or potential CSGs and those that are, to date, somatically mutated in cancer but not implicated in cancer susceptibility (non‐CSG CRGs).

#### 3.2.1 Germline variants in CSGs

7.5% (7 of 93) of participants with MPRT had a heterozygous P/LP variant in a CSG (*n* = 5) associated with a dominantly inherited cancer predisposition disorder (adCSGs) (*VHL* (*n* = 2), *SDHA* (*n* = 2), *CHEK2* (*n* = 1), *MITF* (*n* = 1) and *CDKN2A* (*n* = 1)) (Table 1). A further 9.7% (9/93) of participants with MPRT had a VUS in 9 CSGs (*ALK*, *BMPR1A*, *CHEK2*, *MSH6*, *PALB2*, *POLD1*, *RUNX1*, and *WT1*; see Supplementary Table 4). In addition, heterozygous P/LP variants were also detected in CSGs (*n* = 4) that predispose to cancer when P/LP variants are present biallelically (*NBN* (P/LP = 1), *RECQL4* (P/LP = 1), *MBD4* (P/LP = 1), and *NTHL1* (P/LP = 1), Supplementary Tables 5 and 6).

**TABLE 1:** Participants (*n* = 7) with multiple primary renal tumours (MPRT) with pathogenic or likely pathogenic germline variants in the cancer susceptibility genes with autosomal dominant inheritance.^40^

Gene	Variants	cDNA	Protein	Participant ID	Tumour (age)	Previously reported
*CDKN2A*	chr9:21971200_C/G	c.159G>C	p.Met53Ile	MPRT001	Other RCC (50–55), CCRCC (60–65)	Whitworth et al.^5^
*CHEK2*	chr22:28695238_TA/T	c.1392del	p.Ser465fs	MPRT002	RCC NOS (55–60), RCC NOS (55–60)	Whitworth et al.^5^
*MITF*	chr3:69964940_G/A	c.1273G>A	p.Glu425Lys	MPRT003	CCRCC (75–80), other RCC (75–80)	Yngvadottir et al.^9^
*SDHA*	chr5:256369_TT/−	c.1944_1945del	p.Thr648fs	MPRT004	CCRCC (40–45), CCRCC (NA)	No
				MPRT005	RCC NOS, (30–35) RCC NOS (NA)	No
*VHL*	chr3:10142070_ATCT/A	c.227_229del	p.Phe76del	MPRT006	Other RCC, (35–40) CCRCC (40–45)	Yngvadottir et al.^9^
	chr3:10149874_T/C	c.551 T>C	p.Leu184Pro	MPRT007	CCRCC, (30–35) CCRCC (30–35)	Yngvadottir et al.^9^

*Note*: Variants reported previously in Whitworth et al.^5^ or Yngvadottir et al.,^9^ are indicated The missense variant of CDKN2A was previously reported as variants with uncertain significance (VUS).^5^ CCRCC, clear cell renal cell carcinoma. NOS, not otherwise specified.

Abbreviation: NA, not available.

#### 3.2.2 Germline variants in non‐CSG CRGs

A total of 10 germline high impact pLoF variants were detected in CRGs (*n* = 9) that are somatically mutated in cancer but not currently designated as a CSG (*ASXL1*, *KMT2B*, *KMT2C*, *SH2B3*, *SUZ12*, *MPL*, *SHQ1*, *FGF3*, *TET2*) (Supplementary Table 7). While 5 of these participants had variants in 5 genes (*ASXL1*, *KMT2B*, *KMT2C*, *SH2B3*, and *SUZ12*) for which germline monoallelic P/LP variants are associated with a developmental disorder, no evidence of these conditions was recorded in the available clinical information.

### 3.3 Germline variants in cancer related genes in individuals with MPT:RCC + X

After the application of a series of stringent filters, 432 variants in 216 of 550 CRGs were prioritised. These were divided into two groups according to whether the relevant genes were confirmed or potential CSGs and those that are, to date, non‐CSG CRGs.

#### 3.3.1 Germline variants in CSGs

In 6.1% (27/441) probands with MPT:RCC + X, P/LP variants were detected in 13 known adCSGs including *CHEK2* (*n* = 10), *MITF* (*n* = 3), *BAP1* (*n* = 1), *FH* (*n = 1*), *FLCN* (*n = 2*), *PTEN* (*n = 1*), *SDHA* (*n* = 2), *SDHB* (*n* = 1), *ATM* (*n* = 2), *MLH1* (*n* = 2), *PMS2* (*n* = 1), *RET* (*n* = 1), and *TP53* (*n* = 1) (Tables 2 and 3). A further 8.6% (38/441) of participants with MPT:RCC + X and no P/LP variant in a CSG had a VUS in a RCC‐linked CSG (*BAP1*, *CHEK2*, *FH*, *TSC2*) (see Supplementary Table 8) and in 22 other adCSGs (*ALK*, *APC*, *ATM*, *AXIN2*, *BRCA2*, *BRIP1*, *CBL*, *CEBPA*, *DICER1*, *EGFR*, *KIT*, *MEN1, PALB2*, *PMS2*, *POLD1*, *POLE*, *RAD51D*, *SMARCB1*, *SUFU*, *TERT*, *TMEM127*, *TP53*) that are not typically associated with RCC predisposition (Supplementary Table 9). In addition, heterozygous variants were also detected in 5 participants in 4 CSGs that predispose to cancer when P/LP variants are present biallelically (*BLM* (*n* = 2), *ERCC3* (*n* = 1), *ERCC4* (*n* = 1) and *FANCA* (*n* = 1)) (Supplementary Table 10).

**TABLE 2:** The cancer records of the 21 participants with a renal cell carcinoma (RCC) and an extra‐renal tumours that were identified as carriers of pathogenic or likely pathogenic variants in the RCC‐associated cancer susceptibility genes.

Gene	Tumour susceptibility	Variant	cDNA	Protein	Participant ID	Tumour (age)	Previously reported
*BAP1*	RCC Uveal/cutaneous melanoma, mesothelioma, meningioma (AD)	chr3_52409585_C/T	c.91G>A	p.Glu31Lys	MPT01	Uveal melanoma, (35–40) RCC (35–40)	Whitworth et al.^5^
*CHEK2*	Breast cancer, colorectal cancer, prostate cancer, pancreatic cancer, ovarian cancer, thyroid cancer, RCC (AD)	chr22_28695238_TA/T	c.1392del	p.Ser465Valfs	MPT02	Colorectal (80–85), RCC (80–85)	Yngvadottir et al.^9^
		chr22_28695868_AG/A	c.1229del	p.Thr410Metfs	MPT03	Breast (55–60), RCC (60–65)	No
					MPT04	Chronic myeloid leukaemia (65–70), RCC (60–65)	Whitworth et al.^5^
					MPT05	Colorectal (70–75), lymphoma (70–75), RCC (70–75)	Whitworth et al.^5^
					MPT06	Breast (50–55), colorectal (60–65) ovary (50–55), RCC (60–65)	No
					MPT07	Acute lymphoblastic leukaemia (0–5), RCC (25–30)	No
					MPT08	Breast (60–65), RCC (60–65)	No
					MPT09	Thyroid (40–45), RCC (40–45)	No
					MPT10	Thyroid, (35–40) RCC (60–65)	Yngvadottir et al.^9^
					MPT11	Chronic lymphocytic leukaemia (55–60), RCC (55–60)	Yngvadottir et al.^9^
*FH*	RCC, cutaneous and uterine leiomyomas, rarely uterine leiomyosarcoma, pheochromocytoma, paraganglioma (AD)	chr1_241502552_T/G	c.1127A>C	p.Gln376Pro	MPT12	Thrombocythemia (60–65), RCC (60–65)	Yngvadottir et al.^9^
*FLCN*	Fibrofolliculomas, RCC (AD)	chr17_17224068_TGAA/T	c.469_471del	p.Phe157del	MPT13	Thyroid (60–65), RCC (55–60)	Whitworth et al.^5^
		chr17_17216394_T/TG	c.1285_1286insC	p.His429Profs	MPT14	Ovary (40–45), RCC (40–45)	No
*MITF*	Melanoma (AD) RCC	chr3_69964940_G/A	c.1273G>A	p.Glu425Lys	MPT15	Colorectal (70–75), RCC (65–70)	No
					MPT16	Skin (50–55), RCC (60–65)	Yngvadottir et al.^9^
					MPT17	Breast (60–65), colorectal (60–65), RCC (60–65)	No
*PTEN*	Breast cancer, thyroid cancer, endometrial cancer, colorectal cancer, melanoma, RCC, gastrointestinal hamartomas (AD)	chr10_87957915_C/T	c.697C>T	p.Arg233Ter	MPT18	Uterus, (35–40) thyroid (50–55), RCC (60–65)	Whitworth et al.^5^
*SDHA*	Paraganglioma, pheochromocytoma, gastrointestinal stromal tumour (GIST), RCC (AD)	chr5_223509_C/T	c.91C>T	p.Arg31Ter	MPT19	Breast (70–75), RCC (70–75)	Yngvadottir et al.^9^
					MPT20	Skin (70–75), RCC (75–80)	Yngvadottir et al.^9^
*SDHB*	Paraganglioma, pheochromocytoma, RCC, GIST (AD)	chr1_17024015_C/A	c.600G>T	p.Trp200Cys	MPT21	Testis (40–45), RCC (40–45)	Yngvadottir et al.^9^

*Note*: Variants reported previously in Whitworth et al.^5^ or Yngvadottir et al.,^9^ are indicated.

Abbreviations: AD, autosomal dominant. AR, autosomal recessive.

**TABLE 3:** The cancer records of the seven participants with renal cell carcinoma (RCC) and extra renal tumours that were identified as carriers of pathogenic or likely pathogenic variants in other cancer susceptibility genes with autosomal dominant inheritance.

Gene	Tumour susceptibility	Variant	cDNA	Protein	Participant ID	Tumour (age)
*ATM*	Breast cancer (AD)	chr11_108284280_AG/A	c.3801del	p.Val1268Ter	MPT22	Skin (basal cell carcinoma) (65–70), head/neck (70–75), RCC (65–70)
		chr11_108329202_T/G	c.7271 T>G	p.Val2424Gly	MPT23	Thyroid (55–60), uterus (55–60), head/neck (55–60), RCC (50–55)
*MLH1*	Colorectal cancer, endometrial cancer, ovarian cancer, gastric cancer, small intestinal cancer, urothelial cancer, pancreatic cancer (AD)	chr3_36996698_AC/A	c.197del	p.Ile68Serfs	MPT24	Colorectal (55–60), RCC (55–60)
		chr3_37020356_A/G	c.931A>G	p.Lys311Glu	MPT25	Colorectal (70–75), prostate (70–75), RCC (70–75)
*PMS2*	Colorectal cancer, endometrial cancer and ovarian cancer (AD)	chr7_5986986_CT/C	c.1778del	p.Lys593Serfs	MPT26	Prostate (70–75), RCC (75–80)
*RET*	Medullary thyroid cancer, pheochromocytoma (AD)	chr10_43119548_G/A	c.2410G>A	p.Val804Met	MPT19	Breast (70–75), RCC (70–75)
*TP53*	Breast cancer, sarcoma, lung cancer, adrenocortical carcinoma, glioblastoma, astrocytoma, colorectal cancer (AD)	chr17_7674945_G/A	c.586C>T	p.Arg196Ter	MPT27	Brain (45–50), uterus (45–50), RCC (45–50)

*Note*: Participants MPT34 and MPT36 were reported previously in Whitworth et al. (5)or Yngvadottir et al.^9^

Abbreviations: AD, autosomal dominant. AR, autosomal recessive.

#### 3.3.2 Germline variants in non‐CSG CRGs

A total of 11 germline high‐impact pLoF variants were detected in 8 CRGs that are somatically mutated in cancer but not currently designated as a CSG (*DNMT3A*, *KMT2B*, *PDGFRB*, *SH2B3*, *FANCF*, *FANCI*, *FGF5*, *RAD50*) (Supplementary Table 11). Whilst 7 participants had variants in genes (*DNMT3A*, *KMT2B*, *PDGFRB* and *SH2B3*) for which germline monoallelic P/LP variants are associated with an inherited disorder, no evidence of these conditions was recorded in the available clinical information.

### 3.4 Comparison of germline genetic variants in CSGs in MPRT and MPT:RCC + X and genotype–phenotype correlations

The overall frequencies of P/LP adCSGs variants in participants with MPRT and MPT:RCC + X were 7.5% and 6.1%, respectively, (*p* > .01); however, in participants with MPRT and a RCC diagnosed <66 years, the diagnostic yield was 9.4% (Table 4). In participants with MPT:RCC + X, the diagnostic yield in those with a RCC diagnosed before age 66 years was 10.4%, which was similar to that in participants with both a RCC and an extra‐renal neoplasm diagnosed <66 years (10.3%). Comparison of P/LP CSG frequencies in males and females in MPRT and MPT:RCC + X groups showed that, in general, female participants with MPT (both MPRT and MPT:RCC + X) were more likely to have a genetic cause than male participants (diagnostic yields: MPRT = 11.5% and 6.0% and MPT:RCC + X: 8.7% and 4.5% respectively) though the differences were not statistically significant (*p* > .05). Similarly, the differences in diagnostic yields according to renal histopathology (clear cell versus other) were not statistically significant (MPRT: 6.9% vs. 6.1% and MPT:RCC + X: 5.5% and 4.1%, respectively). As lung cancer, non‐melanoma skin cancer, and cervical cancer are associated with strong environmental factors, we investigated whether excluding MPT:RCC + X participants (*n* = 81) with any one of these cancers would increase diagnostic yield and found a small increase to 6.9% (25/360) and then to 12.9% (19/147) in those with a RCC diagnosed <66 years.

**TABLE 4:** The percentage of pathogenic or likely pathogenic variants in autosomal dominantly inherited cancer susceptibility genes in participants with multiple primary renal tumours (MPRT) and participants with a renal cell carcinoma and an extra‐renal tumour (MPT:RCC + X) in different RCC age groups.

Age of RCC		≤45	≤50	≤55	≤60	≤65
MPRT	*n*	4	4	5	6	6
	%	19.0	11.4	11.9	10.9	9.4
MPT:RCC + X	*n*	6	6	7	12	20
	%	18.8	11	8.6	8.9	10.4
MPT:RCC + X excluding lung cancer, non‐melanoma and cervical cancer	*n*	6	6	7	12	19
	%	27.3	15.0	11.3	11.4	12.8

The relative frequencies of individuals who harboured adCSGs P/LP variants in MPRT and MPT:RCC + X cohorts (based on the variants listed in Tables 1, 2, 3) were compared. Only 1 of 7 (14%) participants with MPRT had a P/LP variant in a CSG (*CDKN2A*) not generally recognised as RCC‐linked CSG, whereas 6/27 (22%) participants with MPT:RCC + X had a P/LP variant in a dominantly inherited CSG not regularly associated with RCC (*ATM*, *MLH1*, *PMS2*, *TP53*). Among participants with MPT:RCC + X, the tumour phenotypes associated with P/LP CSG variants were evaluated according to whether they were typical or atypical for the relevant CSG. For the most commonly mutated CSG, *CHEK2*, 70% (7/10) carriers had an RCC + X phenotype that included a well‐recognised non‐renal tumour associated with *CHEK2*, including breast cancer, colorectal cancer, ovarian cancer, and thyroid cancer, though only 43% (3/7) of the participants had the non‐renal tumours diagnosed before the age of 60 years old. As for the other P/LP variants in RCC‐associated CSG carriers, only one *BAP1* carrier (MPT01) and one *PTEN* carrier (MPT20) had a characteristic associated extra‐renal tumour (uveal melanoma (in MPT01) and endometrial cancer and thyroid cancer (in MPT20) respectively). No typical non‐renal tumours were reported in the participants with *FH*, *FLCN*, *MITF*, *SDHx* P/LP variants (Table 2).

To identify potential clinical characteristics that might be assessed to better identify those in the MPRT and the MPT:RCC + X cohorts that were most likely to have a P/LP CSG variant, receiver operating characteristics (ROC) curve analysis was performed for various age categories for males/females and both combined. For the MPRT cohort, AUC was highest for all cases, males, and females using age categories of age at diagnosis for first RCC diagnosis of ≤45 years (AUC = 0.69), ≤45 years (AUC = 0.77) and ≤60 years (AUC = 0.76), respectively. When analysing age at second RCC, the AUC was highest at ≤40 years (AUC = 0.68), ≤40 years (AUC = 0.72) and ≤65 years (AUC = 0.82), respectively, for all cases, males, and females (Supplementary Table 12).

For MPT:RCC + X cohort, the highest AUCs for all cases, for males and for females were found using age categories of ≤65 years (AUC = 0.66), ≤50 years (AUC = 0.68) and ≤ 65 years (AUC = 0.68), respectively. When the analysis was repeated after excluding participants with lung, cervical or non‐melanoma skin cancer the AUCs for all cases, for males and for females were highest at age categories of ≤65 years (AUC = 0.65), ≤45 years (AUC = 0.61) and ≤ 65 years (AUC = 0.68) (Supplementary Table 12). To investigate whether it was possible to derive ‘MPRT‐CSG’ or ‘MPT:RCC + X CSG’ risk scores that could be used to select patients with these phenotypes for CSG testing, we undertook multivariate analysis using age, sex, RCC histopathology (clear vs. non‐clear), and non‐renal tumour type as variables. Multiple regression did not reveal statistically significant associations for age (*p* = .06), sex (*p* >.1) or histology (*p* >.1). The AUC for this multiple regression model were 0.73 for MPRT and 0.64 for MPT:RCC + X. Hence, in the present study, we did not identify a combination of clinical variables that would have clinical utility for stratifying patients with MPRT/MPT:RCC + X and could be used to prioritise for genetic testing.

## 4 DISCUSSION

We investigated the frequency of germline variants in CSGs in patients with RCC and multiple primary tumours. Though some participants had been included in previous studies^5^, ^9^, ^10^ for this report we reanalysed the genomic data for a much larger number of CRGs and included a detailed analysis of the clinical phenotypes and their relevance for diagnostic yields. We subdivided participants into those with two or more primary renal tumours (MPRT) and those with an RCC and an extra‐renal tumour (MPT:RCC + X). Although we searched for germline variants in a large number of CRGs, in practice, the variants of potential clinical relevance were restricted to confirmed CSGs in which monoallelic variants are known to predispose to cancer (adCSGs). The overall frequencies for P/LP variants in adCSGs in the MPRT and MPT:RCC + X cohorts were 7.5% and 6.1%, respectively. However, this increased to 9.4% and 10.4%, respectively, in those diagnosed with an RCC before age 66 years.

There are some limitations to our study. The diagnostic yield of genomic testing in a MPT cohort will be influenced by the method of ascertainment, the extent of prior genetic testing, and the number of CSGs analysed. Many of the MPRT cohort we studied had undergone prior clinical genetic evaluation and genetic testing for a panel of inherited RCC CSGs (*FH*, *FLCN*, *MET*, *SDHB*, *VHL*) and were therefore likely to be depleted of syndromic cases of MPRT. In contrast, most of the MPT:RCC + X cohort were ascertained following the occurrence of an RCC and recruitment to the 100kGP and had not undergone prior genetic testing. Participants with RCC in the 100kGP study were recruited at the time of renal surgery; therefore, individuals who presented with advanced cancer that were unsuitable for surgery would not have been recruited. Previously, in a cohort of patients with MPT comprising a broad range of tumour types and who had mostly undergone prior genetic testing, potentially pathogenic CSG variants were identified in ~15% of cases and in ~40% of these, the MPT phenotype contained tumours that were not typically associated with that relevant CSG. This suggested that the range of tumours associated with CSGs might be wider than it has been generally recognised. In the current study, in which we focused on two cohorts with MPT that included an RCC, we also found that some participants had variants in CSGs not generally considered to predispose to RCC.

The MPRT and MPT:RCC + X cohorts were heterogeneous for clinical features that can be associated with specific inherited cancer syndromes. Whilst variants in some RCC CSGs are associated with specific RCC histopathology (e.g., clear cell RCC in VHL disease and type 1 papillary RCC with germline *MET* P/LP variants) we found no significant differences in diagnostic yields in both MPRT and MPT:RCC + X cohorts according to histopathology (clear cell versus other subtypes). However, we did find a relative over‐representation of multiple papillary RCC in the MPRT group, which is consistent with a similar observation from a literature series of MPRT cases.^8^ In the MPT:RCC + X cohort, the most striking heterogeneity was in the range of extra‐renal tumour types and diversity of histopathological subtypes within tumours from a single location (e.g., skin). From an inherited RCC syndrome perspective, the significance of an extra‐renal tumour will vary according to the specific tumour type and age at diagnosis (plus family history). For example, certain rare tumours might be characteristic of a specific disorder (e.g., uveal melanoma and BAP1 cancer predisposition syndrome or haemangioblastoma and VHL disease). On the other hand, common cancers (e.g., breast and colorectal cancers) are less specific and might be expected to occur by chance in some patients with RCC, or their occurrence may reflect shared polygenic genetic or environmental risk factors (e.g., smoking, obesity etc.).

In patients with MPT:RCC + X phenotypes, it can be postulated that cancers that are strongly linked to environmental factors (e.g., cutaneous squamous or cutaneous basal cell carcinomas or lung cancers) may be considered to be less significant as an indicator of genetic predisposition. These considerations are reflected in the development of guidelines for eligibility for CSG panel testing for individuals with RCC and other cancers. For example, in the United Kingdom, eligibility criteria for genetic testing in individuals with RCC include the presence of rare tumours such as haemangioblastoma, phaeochromocytoma/paraganglioma, fibrofolliculomas, mesothelioma, or uveal melanoma that are associated with CSGs included in the inherited RCC gene testing panel (*BAP1*, *FH*, *FLCN*, *[MET]*, *SDHB*, *VHL*).^36^ An earlier age at cancer diagnosis is a feature of many inherited cancer predisposition syndromes and is commonly used to assess the likelihood of an underlying genetic cause in a patient with MPT, such that an early age at diagnosis of RCC is an independent eligibility criterion for offering genetic testing in the United Kingdom.^34^ However, the current United Kingdom eligibility criteria for testing patients with MPRT have no upper age limit, though our results suggest that the cost‐effectiveness of genetic testing might be improved by not routinely offering testing to older participants (e.g., ≥66 years). Although it might be argued that further reducing the upper limit of the age at diagnosis of the first RCC for genetic testing would increase cost‐effectiveness (see AUC analysis in Supplementary Table 12) this would increase the number of patients, particularly females, with germline pathogenic variants that would escape detection, and a diagnostic yield of ~10% is widely considered a reasonable result for diagnostic testing. Apart from the features associated with specific inherited RCC predisposition syndromes such as VHL disease, HLRCC, BAP1 cancer predisposition syndrome, PTEN hamartoma syndrome, and with SDH subunit gene mutations (*SDHA/B/C/D*) (e.g., phaeochromocytoma/paraganglioma, wild type gastrointestinal stromal tumour), guidelines for genetic testing in MPT:RCC + X have not been defined.

Previously we have noted that in patients presenting with MPT, germline P/LP variants may be detected not only in CSGs that are typically associated with the MPT cancer types observed but also in other CSGs that are not typically associated with RCC and/or the relevant extra‐renal tumour.^9^, ^10^ Various reasons for this have been suggested, including that the spectrum of tumour types associated with CSGs may be wider than currently recognised and that some associations may be coincidental.^9^, ^10^ Therefore, there is a tension between expanding gene testing panels to detect more germline pathogenic variants in CSGs and increasing the likelihood of incidental findings. Previously we have suggested that if inherited RCC gene panel testing, such as those used in the United Kingdom, is to be expanded, then the most relevant additional genes would be *CHEK2* and additional SDHx sub‐unit genes (*SDHA*, *SDHC*, *SDHD*).^9^ This would apply to MPRT, as the addition of these four genes to the current six CSG panel would increase the diagnostic yield in our MPRT cohort from 3.1% (2/64) to 7.8% (5/64) in those diagnosed with RCC before age 66 years. The genetic architecture of MPT:RCC + X is more heterogeneous than that for MPRT, and it might be argued that in individuals with MPT:RCC + X without features of a specific inherited cancer syndrome, genetic testing should be performed using a large panel of CSGs rather than individualising gene panels according to the specific extrarenal tumours present (the use of a large panel of adCSGs would increase diagnostic yield to 10.4% (20/192) from 2.6% (5/192) and 6.8% (13/192) for 6 and 10 gene RCC panels, respectively, in those diagnosed with RCC before age 66 years).

Despite comprehensive genomic analysis by exome or GS, a genetic cause for MPRT or MPT:RCC + X was identified in only a minority of cases. Negative testing might be caused by a variety of reasons, including (a) somatic mosaicism for a variant, (b) variants in the non‐protein coding region, (c) failure to realise that a CSG VUS is in fact pathogenic, (d) germline pathogenic variants in a novel, as yet unidentified, CSG, (e) polygenic effects, or (f) the MPT resulted from environmental factors, or (g) metastatic disease being misdiagnosed as MPRT or MPT:RCC + X.^8^, ^35^, ^36^ Although we searched for germline variants in ~500 CRGs, we did not identify a novel CSG for MPRT or MPT:RCC + X phenotypes. Interestingly, apparently pathogenic germline loss of function variants were identified in a number of chromatin‐related genes, and RCC is known to harbour frequent somatic mutations in genes regulating chromatin function.^37^ However, many of the genes in which we found variants have been associated with developmental disorders, and there was no evidence of the relevant conditions in the available clinical records. Interestingly, genomic studies on population‐based cohorts have suggested that rare protein‐truncating variants in developmental disorder genes do not always cause a severe phenotype, and in some cases, can be associated with subclinical effects.^38^

In clinical cancer genetics practice eligibility for genetic testing criteria must strike a balance between restrictive criteria that increase diagnostic yield but exclude patients who would have benefited from testing and wider criteria which may reduce cost‐effectiveness. Currently in the NHS England genomic testing eligibility criteria there is no upper limit for testing patients with non‐syndromic MPRT. However, our findings suggest that, in the absence of specific risk factors such as a family history, restricting testing to those whose first RCC was diagnosed before age 66 years would improve cost‐effectiveness of genetic testing and provide a diagnostic yield of ~10%.

For patients with MPT:RCC + X, current eligibility for genetic testing is usually dependent on the presence of rare features of a rare inherited cancer syndrome. However, we found that 26% (7/27) of MPT:RCC + X participants with a P/LP adCSG variant had a more common tumour type that is not specific for an inherited cancer syndrome; these findings suggest that genetic testing using a large panel of CSGs could be offered to patients with MPT:RCC + X diagnosed with RCC before age 66 years. In our analysis, the diagnostic yield for such an approach was 10.4%, and this increased to 12.9% if participants in which the extra‐renal tumour was a lung cancer, non‐melanoma skin cancer, or cervical cancer were excluded (11.0% in those without clinical evidence of VHL disease, HLRCC, BAP1 cancer predisposition syndrome, PTEN hamartoma syndrome or SDH subunit gene mutations).

In conclusion, our findings should prompt further investigations of the genetic basis of MPRT and MPT:RCC + X in large genomically‐characterised cohorts to confirm our findings and enlarge the evidence base for developing agreed criteria for eligibility for genetic testing and gene panel composition for MPRT and MPT:RCC + X. The phenotypic heterogeneity in MPT:RCC + X patients and the frequent apparent discordance between the CSG and the tumour types observed may provide challenges to interpreting the relevance of some genetic findings, but increased application of germline sequencing will facilitate the refinement of clinical diagnostic pathways. In addition, somatic sequencing in multiple tumours in patients with MPRT or MPT:RCC + X phenotypes can help resolve the relevance of uncertain germline findings and also enable selection of cases for targeted therapies (e.g., PARP inhibitors for homologous recombination deficient cancers or immune checkpoint inhibitor therapy in mismatch repair deficient tumours).^35^, ^39^

## AUTHOR CONTRIBUTIONS

**Huairen Zhang:** Investigation; writing – original draft; methodology; formal analysis; data curation. **Bryndis Yngvadottir:** Investigation; writing – review and editing; formal analysis. **Avgi Andreou:** Investigation; writing – review and editing; formal analysis. **Yasemin Cole:** Investigation; writing – review and editing; formal analysis. **Emma R. Woodward:** Writing – review and editing; formal analysis. **James Whitworth:** Investigation; writing – review and editing; formal analysis; supervision. **Eamonn R. Maher:** Conceptualization; writing – original draft; formal analysis; supervision; resources.

## FUNDING INFORMATION

We thank Cambridge NIHR Biomedical Research Centre (BRC‐1215‐20014), Cancer Research UK Cambridge Cancer Centre, and VHL UK/Ireland for research funding. ERW is supported by the National Institute for Health and Care Research (NIHR) Manchester Biomedical Research Centre (BRC) (NIHR203308). The views expressed are those of the authors and not necessarily those of the NIHR or the Department of Health and Social Care. YC is funded by Gates Cambridge Scholarship and the NIH Oxford‐Cambridge Scholars Program.

## CONFLICT OF INTEREST STATEMENT

The authors declare no conflict of interest.

## ETHICS STATEMENT

All participants gave written informed consent to research. The HGD (Molecular Pathology of Human Genetic Disease) study was approved by the South Birmingham Research Ethics Committee (Reference 5175; IRAS 50895) and the 100,000 Genomes Project was approved by the Health Research Authority Committee East of England – Cambridge South (14/EE/1112). The study was performed in accordance with the Declaration of Helsinki.

## Supporting information


**Supplementary Table 1:** The sequencing coverage and quality statistics for each sample.
**Supplementary Table 2**: List of cancer‐related genes studies (*n* = 550).
**Supplementary Table 3**. The mean age at diagnosis of the most common extra.
**Supplementary Table 4**. Participants (*n* = 9) with multiple primary renal tumours.
**Supplementary Table 5**. Participants (*n* = 2) with multiple primary renal tumours.
**Supplementary Table 6**. Participants (*n* = 3) with multiple primary renal tumours.
**Supplementary Table 7**. Participants (*n* = 5) with multiple primary renal tumours.
**Supplementary Table 8**. Participants (*n* = 9) with a renal cell carcinoma (RCC).
**Supplementary Table 9**. Participants (*n* = 35) with a renal cell carcinoma (RCC).
**Supplementary Table 10**. The cancer records of the 5 participants with a renal.
**Supplementary Table 11**. Participants (*n* = 11) with a renal cell carcinoma (RCC).
**Supplementary Table 12**. The area under the curve (AUC) result to compare.

## Data Availability

Data from participants in the 100,000 genomes project used for this study can be accessed through the Genomics England Research Environment. Academic researchers can apply to become a member of the Genomics England Research Network () subject to their institution having signed a participation agreement. Data from HGD‐RCC participants will be made available (subject to ethical considerations) to approved researchers by contacting the corresponding author.
