# Supplementary material for: Clinical and genetic features of multiple primary tumours cohorts with a renal cell carcinoma: Implications for molecular genetic investigations
Source: Int J Cancer. 2025 Aug 22;157(12):2532–43. doi: 10.1002/ijc.70085 (PMC12541567; doi:10.1002/ijc.70085)
Supplement: Supplementary file 1 — Supplementary Table 1: The sequencing coverage and quality statistics for each sample. Supplementary Table 2: List of cancer‐related genes studies (n = 550). Supplementary Table 3. The mean age at diagnosis of the most common extra. Supplementary Table 4. Participants (n = 9) with multiple primary renal tumours. Supplementary Table 5. Participants (n = 2) with multiple primary renal tumours. Supplementary Table 6. Participants (n = 3) with multiple primary renal tumours. Supplementary Table 7. Participants (n = 5) with multiple primary renal tumours. Supplementary Table 8. Participants (n = 9) with a renal cell carcinoma (RCC). Supplementary Table 9. Participants (n = 35) with a renal cell carcinoma (RCC). Supplementary Table 10. The cancer records of the 5 participants with a renal. Supplementary Table 11. Participants (n = 11) with a renal cell carcinoma (RCC). Supplementary Table 12. The area under the curve (AUC) result to compare. [file IJC-157-2532-s001.pdf]

SUPPLEMENTARY INFORMATION

Clinical and genetic features of multiple primary tumours cohorts with a renal cell carcinoma: implications for molecular genetic investigations

Huairen Zhang, Bryndis Yngvadottir, Avgi Andreou, Yasemin Cole, Emma R Woodward, James Whitworth, Eamonn R Maher

Table of Contents:

**Supplementary Methods**	**page 3**
**Supplementary Table 1**	**page 4**
**Supplementary Table 2**	**page 22**
**Supplementary Table 3**	**page 24**
**Supplementary Table 4**	**page 25**
**Supplementary Table 5**	**page 26**
**Supplementary Table 6**	**page 27**
**Supplementary Table 7**	**page 28**
**Supplementary Table 8**	**page 29**
**Supplementary Table 9**	**page 31**
**Supplementary Table 10**	**page 35**
**Supplementary Table 11**	**page 36**
**Supplementary Table 12**	**page 38**

Supplementary Methods

Participants from HGD-RCC study either had exome sequencing with Illumina TruSeq Exome library preparation on the Illumina HiSeq 4000 or Illumina NextSeq platform or genome sequencing with Illumina TruSeq DNA PCR-Free Library Prep Kit on the Illumina HiSeq 2500 sequencer (Smith PS et al. Genes Chromosom Cancer. 2021;60:5–16; Whitworth J et al. Am J Hum Genet. 2018;103:3-18). For participants from 100kGP, genome sequencing was performed with TruSeq PCR-free libraries on the Illumina HiSeq X technology (Yngvadottir B et al. Hum Mol Genet. 2022;31(17):3001–11; Culliford R et al. Nature Communications 2024;15:5935). Details of the sequencing coverage was summarised below.

Supplementary Table 1: The sequencing coverage and quality statistics for each sample.

Sample ID	Total number of sequenced reads	Total number of uniquely mapped non-duplicate reads	Total number of covered bases	Minimum coverage per base	Median coverage per base	Maximum coverage per base	Percentage of targeted bases with coverage ≥ 10
GEL_1	1039719223	792613784	2853993751	0	40	193616	99.4028
GEL_2	874705277	590607356	2834763515	0	29	143024	99.2697
GEL_3	949632260	803330054	2906520522	0	40	110577	98.8757
GEL_4	891514014	762822704	2926347195	0	38	66447	98.8352
GEL_5	996659723	769010100	2906149076	0	38	125581	98.9013
GEL_6	935833706	798387594	2919559869	0	40	77056	98.6486
GEL_7	989607234	785460521	2924657811	0	39	82988	98.9033
GEL_8	966242982	760108842	2925727583	0	38	91709	98.7352
GEL_9	940239240	772303727	2924662029	0	39	87832	98.8932
GEL_10	997139829	822737400	2906328199	0	41	96130	98.9199
GEL_11	910245294	771627165	2926547007	0	39	61861	98.9056
GEL_12	972381028	767189645	2924928041	0	38	103768	98.868
GEL_13	1367975700	1159125972	2926021582	0	59	109602	99.098
GEL_14	1323439318	1142592166	2923708549	0	58	95622	99.0976
GEL_15	889898908	774574273	2925304213	0	39	94032	98.8677
GEL_16	869197638	755872467	2925666459	0	38	88819	98.8149
GEL_17	946984938	771965066	2924332891	0	39	108774	98.9031
GEL_18	889245923	765652376	2906307084	0	38	113265	98.933
GEL_19	1363041717	1179807571	2925483657	0	58	105273	99.1166
GEL_20	1117097205	943527121	2925737981	0	48	107841	99.0741
GEL_21	1067901726	875782752	2924289576	0	44	112556	98.926
GEL_22	1123582128	958663647	2924497233	0	49	65612	98.9859

GEL_23	1418612792	1185440723	2925060806	0	60	112094	99.1534
GEL_24	797629266	686097597	2904350915	0	34	108537	98.687
GEL_25	1399811020	1195464200	2905624503	0	60	138156	99.0777
GEL_26	1473169713	1192885867	2925802590	0	60	106053	99.1778
GEL_27	925929823	769453941	2924232469	0	39	81670	98.7577
GEL_28	964720493	713016478	2906000447	0	35	105443	98.8758
GEL_29	2735150151	2272309645	2928627883	0	117	123608	99.3862
GEL_30	1368684184	1156847087	2924079116	0	59	92447	99.0192
GEL_31	904568768	737492218	2923810026	0	37	115268	98.4534
GEL_32	1180601748	1008634905	2906139457	0	51	127713	98.9092
GEL_33	799590452	662514995	2922160752	0	34	69062	98.3432
GEL_34	815036706	673042894	2924445370	0	34	113183	98.4251
GEL_35	908825531	773417322	2923219943	0	39	90568	98.7993
GEL_36	805359196	695229244	2923497217	0	35	95519	98.4916
GEL_37	879843341	750918903	2923737448	0	38	102645	98.7589
GEL_38	1255412293	1049724427	2906386707	0	53	121726	99.0505
GEL_39	1316858139	1123195019	2925538318	0	57	107532	98.4598
GEL_40	1471775649	1247532829	2925197943	0	63	117852	99.1752
GEL_41	1024967853	886536162	2926426643	0	45	105965	98.9342
GEL_42	1213092058	1022849562	2926350137	0	52	92929	99.0275
GEL_43	1325965982	1130786612	2926378363	0	57	114268	99.1225
GEL_44	1165051609	998081512	2907497654	0	50	110144	98.9251
GEL_45	870456320	748925578	2920426777	0	37	73491	98.6275
GEL_46	1306104468	1149394967	2924491872	0	58	112270	99.1156
GEL_47	861754944	743799912	2922927901	0	37	99862	98.7322
GEL_48	1423656896	1217831651	2926537650	0	62	141571	99.1607
GEL_49	867334133	736754502	2903270169	0	37	94404	98.7609

GEL_50	905871597	765470266	2923288689	0	38	87666	98.7308
GEL_51	827811654	720103162	2924418478	0	36	117171	98.6754
GEL_52	1219343951	1048825368	2925578485	0	53	134970	99.0694
GEL_53	799310153	678666194	2923838901	0	34	104679	98.3693
GEL_54	902252987	771592511	2924064256	0	39	126037	98.7281
GEL_55	981090778	841993183	2905309781	0	42	80659	98.8054
GEL_56	857668773	739611000	2903452146	0	37	103920	98.7879
GEL_57	3245290437	2609193195	2908875365	0	133	143495	99.3293
GEL_58	924308840	785721996	2904803208	0	39	108436	98.7884
GEL_59	924308840	785721996	2904803208	0	39	108436	98.7884
GEL_60	924308840	785721996	2904803208	0	39	108436	98.7884
GEL_61	901542608	682278568	2835315115	0	34	169484	99.3821
GEL_62	896236109	698417181	2854067042	0	35	176135	99.2105
GEL_63	865954718	638963041	2853389618	0	32	163822	98.9723
GEL_64	874705277	590607356	2834763515	0	29	143024	99.2697
GEL_65	926861329	736803388	2925773928	0	37	78185	98.8097
GEL_66	880684754	739253079	2927028414	0	37	56828	98.8062
GEL_67	994279727	828198189	2835935897	0	42	182425	99.4727
GEL_68	963637989	781824216	2924366468	0	39	57552	98.8906
GEL_69	921436175	783590342	2924785936	0	39	80767	98.8424
GEL_70	932597289	761541354	2906082864	0	38	108450	98.8628
GEL_71	878745908	763325995	2906784560	0	38	46108	98.8824
GEL_72	976926444	827323421	2928502189	0	41	98669	98.9504
GEL_73	941755145	760478608	2907282667	0	38	90691	98.8689
GEL_74	897823800	662756494	2925710898	0	33	85979	98.4677
GEL_75	900642679	740394236	2906457986	0	37	87422	98.8549
GEL_76	937598941	767574481	2926004784	0	38	56179	98.8879

GEL_77	945904080	773787628	2926162204	0	39	74111	98.898
GEL_78	900684138	750816261	2926549315	0	37	100873	98.7844
GEL_79	980054204	721714604	2906228629	0	36	125909	98.8576
GEL_80	973418476	816916510	2907936307	0	41	81269	98.9339
GEL_81	870387165	758276599	2905776806	0	38	111194	98.8882
GEL_82	986059609	784369385	2926455323	0	39	112096	98.9081
GEL_83	949834071	797279467	2925706130	0	40	98029	98.8979
GEL_84	959653311	815322886	2926211905	0	40	89534	98.8945
GEL_85	838329220	714520910	2905013502	0	36	94112	98.6703
GEL_86	945011274	795117270	2926439366	0	39	98082	98.8805
GEL_87	961716910	774305849	2925703349	0	39	84894	98.8771
GEL_88	990308485	726174027	2907430698	0	36	81648	98.813
GEL_89	949123153	746559267	2923951888	0	37	125955	98.8093
GEL_90	916784382	799398525	2925594324	0	40	106150	98.8893
GEL_91	930775933	721657096	2925068956	0	36	123663	98.7566
GEL_92	971748690	655802068	2905401537	0	32	96040	98.8493
GEL_93	956330419	810420235	2905988000	0	40	115103	98.9001
GEL_94	909632489	770530874	2926043503	0	39	103342	98.8925
GEL_95	962901956	797826182	2906877422	0	40	107028	98.9293
GEL_96	892357657	748342247	2906354940	0	37	115155	98.8837
GEL_97	929506321	779011076	2927050433	0	39	96541	98.882
GEL_98	976636969	706615172	2906114386	0	35	94584	98.8689
GEL_99	962533533	702757190	2905404742	0	35	94707	98.7763
GEL_100	821932703	711600943	2904175106	0	36	121395	98.7309
GEL_101	942912917	779559710	2906609726	0	39	91898	98.8627
GEL_102	913081033	746161795	2926495687	0	37	97845	98.8215
GEL_103	872326287	755550580	2926249356	0	38	98840	98.7651

GEL_104	895669237	749631653	2925412071	0	37	87077	98.8597
GEL_105	912570159	725686166	2924420570	0	36	104781	98.7906
GEL_106	865903428	761097161	2903831473	0	38	110528	98.7728
GEL_107	980939608	837809805	2905432024	0	42	109224	98.7763
GEL_108	861763703	739213594	2904908302	0	37	99320	98.6967
GEL_109	966443514	784209871	2926680137	0	39	89241	98.8628
GEL_110	954366365	774380084	2906506933	0	38	103314	98.9211
GEL_111	946739427	778921676	2925896546	0	39	90430	98.8799
GEL_112	952067602	784676600	2925276210	0	39	127851	98.8441
GEL_113	883204522	774628497	2906858728	0	38	88679	98.8832
GEL_114	956197492	812651883	2926053679	0	41	115438	98.8994
GEL_115	955782151	816956556	2906046735	0	41	116671	98.9106
GEL_116	967770134	802112488	2906195796	0	40	99535	98.9493
GEL_117	943231502	742891278	2925495003	0	37	88913	98.819
GEL_118	981129362	762362106	2906541797	0	38	67565	98.8493
GEL_119	977430665	785014398	2924309322	0	40	111131	98.8399
GEL_120	971240757	777780442	2924799147	0	39	91483	98.8857
GEL_121	837396212	672829699	2904615350	0	34	74034	98.7493
GEL_122	1640151664	1388074842	2929572761	0	70	78382	99.3245
GEL_123	957112799	740399842	2998171572	0	37	166010	98.8525
GEL_124	957112799	740399842	2998171572	0	37	166010	98.8525
GEL_125	969465028	762602552	2906477474	0	38	112503	98.9258
GEL_126	947133710	788473389	2925987006	0	40	97178	98.9193
GEL_127	958980371	677800921	2926161201	0	34	80174	98.6329
GEL_128	980716617	708227119	2905903727	0	35	82324	98.8941
GEL_129	963999133	757705566	2926090361	0	38	91496	98.8378
GEL_130	847212008	666138796	2926214965	0	33	82815	98.5142

GEL_131	967800023	737411678	2925177956	0	37	88207	98.6764
GEL_132	942144893	806626128	3017908602	0	41	154925	98.7789
GEL_133	942144893	806626128	3017908602	0	41	154925	98.7789
GEL_134	915225385	752935147	2925483273	0	38	86259	98.8368
GEL_135	848748732	663177929	2905548912	0	33	83664	98.8133
GEL_136	977438449	788929911	2925185752	0	40	112282	98.9206
GEL_137	910245294	771627165	2926547007	0	39	61861	98.9056
GEL_138	1001600165	822519885	3018731761	0	41	177863	98.9002
GEL_139	1001600165	822519885	3018731761	0	41	177863	98.9002
GEL_140	930992314	752464274	2924404873	0	38	76468	98.8624
GEL_141	952900463	670789534	2925004767	0	34	100408	98.5748
GEL_142	939048503	781488317	2925788998	0	39	93420	98.8892
GEL_143	946476508	780090352	2925901718	0	39	94564	98.8986
GEL_144	924936742	746727538	2925069520	0	37	90989	98.8366
GEL_145	900626348	764664569	2923180626	0	38	71443	98.8321
GEL_146	1323439318	1142592166	2923708549	0	58	95622	99.0976
GEL_147	822771728	703101244	2925692895	0	35	105921	98.6808
GEL_148	826740256	700137316	2905197594	0	35	96811	98.8588
GEL_149	948113775	764558837	2926499464	0	38	103997	98.8924
GEL_150	916970398	798647282	2922919724	0	40	95018	98.8341
GEL_151	978378327	837830406	2925379310	0	42	105912	98.8885
GEL_152	962729783	836355132	2923956307	0	42	93041	98.8857
GEL_153	851634062	752483488	2903750410	0	38	90197	98.7328
GEL_154	878486807	762843510	2922589915	0	38	87817	98.758
GEL_155	927826224	809247741	2923086256	0	41	101292	98.8285
GEL_156	903500868	744504107	2905544421	0	37	100333	98.8654
GEL_157	876993157	709151669	2925149847	0	36	67828	98.7156

GEL_158	909096408	730312618	2925225662	0	37	81541	98.7675
GEL_159	885829940	742010274	2905458798	0	37	71367	98.8757
GEL_160	1183072630	999191664	2908530216	0	50	153550	98.9428
GEL_161	830909300	728747247	3019185289	0	37	164392	98.5837
GEL_162	830909300	728747247	3019185289	0	37	164392	98.5837
GEL_163	1210379645	1059232582	2905343531	0	53	98283	98.9834
GEL_164	886526042	779549308	2903776204	0	39	66807	98.7674
GEL_165	840668424	736741122	2922279895	0	37	123815	98.7009
GEL_166	1430200809	1191876416	2924392596	0	61	125272	99.1465
GEL_167	1164601414	1006707335	2925356812	0	51	65027	99.0513
GEL_168	1536618037	1328352369	2907519363	0	67	81016	99.1254
GEL_169	935493929	767197458	2906276300	0	38	117638	98.8766
GEL_170	889898908	774574273	2925304213	0	39	94032	98.8677
GEL_171	949720898	771404642	2905849231	0	38	89950	98.9263
GEL_172	869197638	755872467	2925666459	0	38	88819	98.8149
GEL_173	966254243	769662566	2925656845	0	39	75797	98.8863
GEL_174	934686079	771050124	2926397368	0	39	111694	98.8968
GEL_175	920555996	766135170	2926502977	0	38	81479	98.9053
GEL_176	951399654	776297119	2926345196	0	39	76330	98.847
GEL_177	939844520	760279712	2926931354	0	38	75108	98.8708
GEL_178	917650805	780579547	2925199598	0	39	111612	98.8762
GEL_179	907787939	789377442	2924312145	0	40	104092	98.8434
GEL_180	863463278	762358763	2922949456	0	39	111160	98.7505
GEL_181	903109040	787064280	2924754105	0	40	95814	98.8309
GEL_182	813218959	709855523	2923232635	0	36	106491	98.5778
GEL_183	862892293	750521703	2924583365	0	38	115013	98.7134
GEL_184	880223745	769179640	2922922117	0	39	89106	98.7855

GEL_185	1096594309	950982036	2925774794	0	48	117821	98.9393
GEL_186	801749589	698927935	2923812632	0	35	74428	98.5787
GEL_187	1167405116	1011401328	2925172224	0	51	95756	99.096
GEL_188	817255657	687658684	3019417550	0	34	193278	98.4754
GEL_189	817255657	687658684	3019417550	0	34	193278	98.4754
GEL_190	978413357	764459718	2924702661	0	38	86378	98.8874
GEL_191	1454873709	1258761406	2925746342	0	64	116052	99.1772
GEL_192	1503758133	1283823107	2905747634	0	65	75147	99.1372
GEL_193	1365969195	1170990997	2926527246	0	59	103811	99.1992
GEL_194	871528556	728142980	2924766343	0	36	110581	98.6976
GEL_195	806666094	676047487	2925062930	0	34	99421	98.6296
GEL_196	822187461	723321883	2924272039	0	36	93994	98.6223
GEL_197	834809115	729537274	2922163470	0	37	88402	98.6677
GEL_198	867116698	741470981	2924425092	0	37	56077	98.7196
GEL_199	972667465	848135506	2924565755	0	43	103021	98.8525
GEL_200	1439057519	1239625784	2926488397	0	63	101458	99.2
GEL_201	1218213569	1102249015	3019909439	0	56	217632	99.1092
GEL_202	1218213569	1102249015	3019909439	0	56	217632	99.1092
GEL_203	1441236467	1241760701	2906203951	0	63	80667	99.1366
GEL_204	1671493545	1399837039	2907339499	0	71	149543	99.0792
GEL_205	801529245	682503137	2922050406	0	34	58718	98.4635
GEL_206	834628181	713669075	2903731934	0	36	124232	98.7619
GEL_207	1343283451	1153189391	2924897196	0	59	142271	99.1165
GEL_208	926363109	791083314	2905636004	0	40	115877	98.7834
GEL_209	854767172	720670809	2923229226	0	36	108517	98.6094
GEL_210	840026767	713722327	2904917611	0	36	131559	98.7159
GEL_211	917828085	780904969	2924291351	0	39	93219	98.7011

GEL_212	1473816757	1250735693	2925792483	0	63	125905	99.1643
GEL_213	1427383899	1213855407	2905538604	0	61	135734	99.0768
GEL_214	1481990305	1258096576	2926699860	0	64	131608	99.2006
GEL_215	1456242495	1254482849	2907343818	0	63	135444	99.1001
GEL_216	1382993498	1206916708	2925012619	0	62	96854	99.1298
GEL_217	976909638	736714150	2925477336	0	37	102019	98.8178
GEL_218	1377457830	1176603996	2925464143	0	60	97259	99.1425
GEL_219	800373427	675629093	2922493382	0	34	95836	98.4848
GEL_220	810516035	693689950	2923115220	0	35	100336	98.5735
GEL_221	811306826	689410878	2904544508	0	35	94821	98.6913
GEL_222	1032419826	874593801	2924934527	0	44	92773	98.8968
GEL_223	926257704	726860055	2924761228	0	36	89655	98.7947
GEL_224	987044222	837915808	2923864366	0	42	95633	98.9315
GEL_225	1117097205	943527121	2925737981	0	48	107841	99.0741
GEL_226	1642814620	1369234125	2925962259	0	69	101517	99.2464
GEL_227	1074533610	923109554	2925183180	0	47	97254	98.9969
GEL_228	1067901726	875782752	2924289576	0	44	112556	98.926
GEL_229	1123582128	958663647	2924497233	0	49	65612	98.9859
GEL_230	941133404	812245295	2903636422	0	41	93618	98.8123
GEL_231	973329020	814872394	2904839031	0	41	93100	98.8719
GEL_232	1323877907	1138181944	2926464997	0	58	108085	99.1245
GEL_233	797629266	686097597	2904350915	0	34	108537	98.687
GEL_234	1501006623	1234613726	2927006283	0	63	149343	99.1783
GEL_235	1481311402	1222656672	2905768196	0	62	142038	99.1075
GEL_236	992764648	718380278	2924649252	0	36	80995	98.8065
GEL_237	873822203	754310441	2904978511	0	38	102165	98.7843
GEL_238	1357481028	1126255020	2924826065	0	57	106678	99.127

GEL_239	925929823	769453941	2924232469	0	39	81670	98.7577
GEL_240	950085780	808316786	2924157337	0	41	79630	98.8531
GEL_241	1502895913	1265855148	2927182208	0	65	122995	98.8747
GEL_242	1246358866	1039148239	2926031719	0	53	132572	99.0131
GEL_243	895470210	774420177	2904230209	0	39	118738	98.8373
GEL_244	796497303	693567270	2922718593	0	35	111083	98.5822
GEL_245	1391684762	1167265709	2905648024	0	59	128072	99.0306
GEL_246	1351127252	1046061383	2925073720	0	53	114004	99.0641
GEL_247	1422734487	1167733589	2907672632	0	59	115295	99.0392
GEL_248	945260444	704193597	2903659169	0	35	100827	98.9026
GEL_249	1127300835	972024753	2906158782	0	49	149987	98.9822
GEL_250	1090262662	932661892	2906592265	0	47	95929	98.9348
GEL_251	1137695711	967435593	2924550335	0	49	103379	99.0459
GEL_252	1019097178	871274795	2925098343	0	44	103182	98.9335
GEL_253	807869257	678150305	2903927304	0	34	99848	98.703
GEL_254	918332686	772893747	2923630101	0	39	93006	98.8204
GEL_255	825786295	701723587	2922897414	0	35	113502	98.603
GEL_256	890781710	758586385	2923137466	0	39	82616	98.6759
GEL_257	1429635252	1172225170	2925988597	0	60	138939	99.1537
GEL_258	795174298	671843499	2923102941	0	34	97226	98.4944
GEL_259	1130606059	988120972	2905730289	0	50	118484	98.9617
GEL_260	952360060	809650791	2906754845	0	41	119218	98.7814
GEL_261	964720493	713016478	2906000447	0	35	105443	98.8758
GEL_262	921896971	680051108	2923163842	0	34	80685	98.5974
GEL_263	940636020	781716911	2904678270	0	39	91381	98.8085
GEL_264	1082007895	916965415	2905656447	0	46	107676	98.8991
GEL_265	835754320	695050590	2922949866	0	35	93981	98.523

GEL_266	1160756954	959830935	2924413451	0	49	137601	99.019
GEL_267	981017262	825396374	2905667390	0	41	143641	98.8493
GEL_268	1055767108	892112026	2906087152	0	45	94449	98.9231
GEL_269	2727254729	2259967063	2928374080	0	116	151155	99.3659
GEL_270	2828548686	2326123582	2910291115	0	118	161544	99.3013
GEL_271	2666346683	2146661984	2908981169	0	109	238306	99.2653
GEL_272	2707277040	2238894462	2910487491	0	113	230627	99.2812
GEL_273	1426405584	1194506896	2923985281	0	60	98760	98.8188
GEL_274	1354934799	1130809982	2905962427	0	57	116449	99.0016
GEL_275	1409672075	1220281118	2905581590	0	62	117322	98.9683
GEL_276	1354913821	1131496158	2905193858	0	57	134321	99.0586
GEL_277	841781001	721138980	2922792293	0	36	133664	98.6557
GEL_278	1097551573	933820970	2906485234	0	47	93066	98.9419
GEL_279	904772583	768994117	2923352981	0	39	101259	98.7563
GEL_280	883973197	745333748	2922939923	0	37	99274	98.7529
GEL_281	838115390	704589135	2903860004	0	35	92919	98.7266
GEL_282	856940917	715623051	2922675809	0	36	71332	98.5411
GEL_283	838642211	706387410	2923202415	0	36	90151	98.649
GEL_284	1494433801	1221574782	2907677272	0	62	130271	99.1018
GEL_285	1503192600	1267858428	2926084856	0	64	118044	99.196
GEL_286	1429692833	1213152130	2925658942	0	62	113235	99.1355
GEL_287	1519004244	1272065941	2926532375	0	65	98840	99.1756
GEL_288	904568768	737492218	2923810026	0	37	115268	98.4534
GEL_289	1402378985	993695363	2925615170	0	50	96814	99.0916
GEL_290	999446123	856232360	2907175860	0	43	65707	98.8922
GEL_291	869757793	733982794	2921992609	0	37	60998	98.7419
GEL_292	962546849	684038270	2904436840	0	34	103623	98.883

GEL_293	925568046	795760867	2903802822	0	40	85570	98.8209
GEL_294	869626354	747757850	2902351476	0	38	97492	98.7872
GEL_295	817370332	680405119	2903645579	0	34	69369	98.6836
GEL_296	892116042	740728324	2922589340	0	37	76029	98.7334
GEL_297	858626389	726284862	2923468587	0	37	119239	98.667
GEL_298	1013129253	870603883	2906116913	0	44	65774	98.8497
GEL_299	956077216	786626906	2925574068	0	39	106958	98.7869
GEL_300	1006263300	829698137	2906306104	0	42	61322	98.8807
GEL_301	863056745	751241411	2904214879	0	38	62487	98.7623
GEL_302	1481982066	1193415492	2926693840	0	61	126590	99.1662
GEL_303	879057088	754755098	2924324109	0	38	95231	98.7522
GEL_304	908621300	771629421	2923813655	0	39	106066	98.7827
GEL_305	1453269809	1194636690	2907707483	0	61	104221	99.0598
GEL_306	1153417117	975825331	2922159137	0	49	126446	99.0473
GEL_307	855510626	731357850	2922475685	0	37	93974	98.6309
GEL_308	816749476	688137740	2922807895	0	35	75233	98.4855
GEL_309	806145186	695702370	2903917899	0	35	71977	98.6895
GEL_310	875910640	754144395	2923174859	0	38	71236	98.719
GEL_311	919772639	772174919	2923230191	0	39	113144	98.7601
GEL_312	884387656	751607690	2922788193	0	38	106998	98.7065
GEL_313	904155492	768131355	2923694979	0	39	74278	98.7431
GEL_314	802164332	680729954	2904382940	0	34	72054	98.6631
GEL_315	834338183	710626431	2903552252	0	36	127003	98.7348
GEL_316	830718279	716806940	2902773571	0	36	116705	98.7679
GEL_317	917135355	798982497	2924251579	0	40	94789	98.7017
GEL_318	851184536	741604398	2903127326	0	37	94505	98.7399
GEL_319	1008222334	868920768	2904440354	0	44	105200	98.8528

GEL_320	1207371159	987499088	2924774224	0	50	135004	99.0055
GEL_321	1497011173	1245686068	2927260652	0	64	112197	99.1379
GEL_322	890114672	761166613	2906252526	0	38	96506	98.72
GEL_323	946778048	788914716	2904901216	0	40	67714	98.808
GEL_324	1570035295	1275526028	2924717804	0	64	128900	99.1675
GEL_325	1374021619	1154504380	2905437259	0	58	109885	99.034
GEL_326	823160648	722536204	2902896480	0	36	54004	98.7166
GEL_327	851140970	728383596	2922846566	0	37	58583	98.6628
GEL_328	907893003	776335881	2922790060	0	39	104075	98.7751
GEL_329	836354942	718580844	2924073248	0	36	96146	98.6328
GEL_330	889068398	759463819	2905368736	0	38	80859	98.7846
GEL_331	832342819	710503085	2924403804	0	36	104500	98.6045
GEL_332	845576247	730399850	2924192395	0	37	61274	98.6494
GEL_333	1301912384	1092251648	2925881549	0	56	114970	99.0939
GEL_334	1393159707	1185186144	2923951750	0	60	148113	99.1603
GEL_335	1135884761	991111986	2925339529	0	50	112846	99.0454
GEL_336	1184592342	998996238	2925059642	0	51	100373	99.0845
GEL_337	1159715530	977760018	2924889573	0	50	117039	99.0641
GEL_338	1255412293	1049724427	2906386707	0	53	121726	99.0505
GEL_339	984642356	836884480	2925084788	0	42	100798	98.8953
GEL_340	1316858139	1123195019	2925538318	0	57	107532	98.4598
GEL_341	825607189	719339916	2923579459	0	36	94429	98.5545
GEL_342	1601526890	1324799763	2925876809	0	67	145613	99.224
GEL_343	904766368	772420501	2923577875	0	39	72673	98.76
GEL_344	870191607	725104833	2924288292	0	37	76582	98.6628
GEL_345	984854912	837280162	2902469024	0	42	70376	98.7709
GEL_346	850731723	737657936	2904426220	0	37	77529	98.8116

GEL_347	1278490890	1088937418	2924715887	0	55	129780	99.0917
GEL_348	1052929188	905894742	2906089959	0	46	99513	98.8927
GEL_349	1157392701	982989926	2908218481	0	49	111787	98.9618
GEL_350	1563294362	1253116408	2926242714	0	64	123649	99.1992
GEL_351	1513886320	1271439683	2926339999	0	65	149875	99.2002
GEL_352	837009022	712154638	2923110292	0	36	89569	98.6138
GEL_353	857974153	728350794	2922278120	0	37	64733	98.6553
GEL_354	844954880	711475690	2922687195	0	36	97791	98.5714
GEL_355	870975010	745683485	2923838206	0	37	115511	98.709
GEL_356	819631000	705316882	2922363913	0	35	80752	98.4788
GEL_357	1433839375	1204932246	2925764137	0	61	104817	99.2123
GEL_358	797238230	688209064	2922788561	0	35	118656	98.4449
GEL_359	1458991956	1232745301	2924815889	0	63	120559	98.9187
GEL_360	1465866858	1277390181	2925873313	0	65	154214	99.1787
GEL_361	1386603330	1189457796	2925891478	0	61	69793	99.105
GEL_362	795440010	691245442	2904177793	0	35	106675	98.6162
GEL_363	893324834	771092099	2925752355	0	39	125322	98.7284
GEL_364	1168461975	1002425674	2926825604	0	51	114635	98.9938
GEL_365	873326092	757612527	2924640257	0	38	111310	98.7406
GEL_366	843823499	730806068	2923767158	0	37	99801	98.6581
GEL_367	1283218334	1099568197	2907988150	0	55	72073	98.9995
GEL_368	1411153419	1167666127	2924485036	0	59	142256	99.1345
GEL_369	843465705	727370780	2922914295	0	37	65662	98.6632
GEL_370	1506634192	1163936300	2905309643	0	58	82637	99.0971
GEL_371	939560380	808436942	2906507519	0	41	102508	98.8634
GEL_372	866248859	734265004	2906309357	0	37	44582	98.7351
GEL_373	843668670	736978127	2903298284	0	37	82619	98.6806

GEL_374	819084114	714579526	2923479697	0	36	83877	98.5177
GEL_375	893196538	768508215	2904481751	0	39	114545	98.8227
GEL_376	825067651	706865786	2923748312	0	35	53689	98.4896
GEL_377	888250989	770746731	2903894017	0	39	88743	98.8026
GEL_378	1094329580	872912434	2923794147	0	44	90256	98.8756
GEL_379	1414888460	1216337062	2908391029	0	61	102654	99.0433
GEL_380	832839386	718889673	2921756511	0	36	70059	98.4802
GEL_381	1328231900	1134059924	2925577533	0	58	108466	99.0801
GEL_382	801373734	677967991	2922177789	0	34	124404	98.4068
GEL_383	1041141465	879448422	2925246286	0	44	125863	98.9183
GEL_384	1041026679	871656051	2906617945	0	44	116001	98.8528
GEL_385	1327947619	1123159784	2908506557	0	56	132534	98.9761
GEL_386	897438969	775499524	2905222245	0	39	120266	98.7921
GEL_387	1189425032	1017086323	2906088870	0	51	140244	98.9508
GEL_388	1349048923	1150788106	2906528838	0	58	148285	98.9931
GEL_389	1327199763	1126332756	2906887819	0	56	134718	98.9892
GEL_390	955137881	815415802	2922964065	0	41	102598	98.79
GEL_391	835887220	716440048	2923375895	0	36	106698	98.5708
GEL_392	851469241	732114174	2904229832	0	37	88340	98.7709
GEL_393	835764357	712622277	2922889028	0	36	113327	98.5962
GEL_394	804150592	698644158	2903609728	0	35	107812	98.7497
GEL_395	1004625862	870426850	2906321214	0	44	130735	98.8591
GEL_396	870456320	748925578	2920426777	0	37	73491	98.6275
GEL_397	1306104468	1149394967	2924491872	0	58	112270	99.1156
GEL_398	795707333	683933738	2903661943	0	34	57732	98.7013
GEL_399	1477783534	1243105347	2906295010	0	63	125416	99.069
GEL_400	1353983625	1160470339	2926854431	0	59	134081	99.1262

GEL_401	1006963442	867662946	2924323154	0	44	85845	98.9639
GEL_402	3045279652	2666031119	2905599246	0	137	121706	99.1469
GEL_403	891862692	766435316	2904078818	0	38	53328	98.7347
GEL_404	820873490	703328897	2903053215	0	35	90705	98.657
GEL_405	930859860	798923995	2923340766	0	40	116776	98.7924
GEL_406	814961431	702550253	2922032498	0	35	75004	98.5924
GEL_407	3385339777	2642616880	2929549867	0	135	228680	99.4155
GEL_408	1358171869	838867842	2922971005	0	41	73259	98.8512
GEL_409	796340786	685191243	2922985129	0	34	86119	98.5337
GEL_410	838411607	728475576	2923537748	0	37	82587	98.6232
GEL_411	835875724	719261762	2921971389	0	36	82880	98.5934
GEL_412	800788037	692023634	2923059196	0	35	74926	98.4882
GEL_413	890414363	751622425	2924384157	0	38	130065	98.6993
GEL_414	889978858	757607495	2904432909	0	38	84942	98.7699
GEL_415	820741442	703460237	2923569316	0	35	118857	98.5492
GEL_416	878468098	756025604	2924642235	0	38	94209	98.7288
GEL_417	1182213656	1004939806	2914610493	0	42	84049	98.3811
GEL_418	777313942	672965175	2905513215	0	34	87958	98.6124
GEL_419	827173437	715911397	2923748651	0	36	93523	98.562
GEL_420	802582624	698222505	2905271398	0	35	96765	98.6382
GEL_421	898797877	785498036	2903853674	0	39	88212	98.819
GEL_422	855068660	743269089	2905237985	0	37	139497	98.7082
GEL_423	1399615783	1192846230	2907302291	0	60	109009	99.0815
GEL_424	1452445779	1229105629	2925967496	0	63	122775	99.154
GEL_425	848462867	715070716	2923610894	0	36	100518	98.689
GEL_426	861768343	719271150	2922900206	0	36	117855	98.6121
GEL_427	823808566	694058653	2923597252	0	35	101119	98.4999

GEL_428	1323431385	1161723063	2924997638	0	59	85842	99.1337
GEL_429	1521417575	1007038198	2906661476	0	50	100112	99.0165
GEL_430	1329564867	1161766210	2907830771	0	59	118606	99.0636
GEL_431	1075989313	937489312	2925250567	0	47	98288	98.9849
GEL_432	1136974235	989229064	2925200914	0	50	131722	99.0774
GEL_433	974530112	824421148	2904978540	0	41	90194	98.8503
GEL_434	885627620	775356316	2923042068	0	39	90287	98.7654
GEL_435	816093316	703442661	2922858112	0	35	90682	98.317
GEL_436	838748063	728264460	2923305412	0	36	100932	98.6652
GEL_437	860921319	730044703	2923747277	0	37	102970	98.648
GEL_438	1303326592	1116996333	2927406273	0	57	161084	98.9847
GEL_439	817534706	702411087	2902645980	0	35	95791	98.7392
GEL_440	843553673	716805983	2923818179	0	36	70238	98.5616
GEL_441	1460961621	1240543476	2925174062	0	63	116402	99.15
GEL_442	1602792648	1394665616	2925066183	0	71	136297	99.2301
GEL_443	846561090	723816904	2923459550	0	37	86159	98.6825
GEL_444	802577659	687466874	2923746093	0	35	85887	98.5183
GEL_445	827613918	710988862	2923478320	0	36	88627	98.6176
GEL_446	1398989960	1198458575	2925231020	0	61	122977	99.1526
HumGenDis_1	167214716	136223775	4860939760	0	95	1217	95.3252
HumGenDis_2	101791666	89554474	3323203911	0	64	1693	94.2314
HumGenDis_3	109179758	94423437	3424058359	0	67	745	93.8143
HumGenDis_4	87101762	74138506	2744962320	0	53	686	92.3383
HumGenDis_5	153759214	121127240	4903136446	0	96	1120	95.6759
HumGenDis_6	139179796	115457188	4281957732	0	85	878	95.2246
HumGenDis_7	116667404	104308569	3702022078	0	72	872	94.4156
HumGenDis_8	154150262	136905799	4678462780	0	92	1201	95.6302

HumGenDis_9	108969608	77563544	3036923412	0	59	726	93.515
HumGenDis_10	102315986	91797348	2975095456	0	58	862	93.1004
HumGenDis_11	113821412	96606775	3268614904	0	64	738	93.9645
HumGenDis_12	107492888	95167832	3731131098	0	70	798	94.3482
HumGenDis_13	136902642	98981451	3828544625	0	77	886	95.1066
HumGenDis_14	121096152	105026621	3213625021	0	62	637	92.5502
HumGenDis_15	103317156	87117593	3038584782	0	62	1367	89.2551
HumGenDis_16	122033072	95445674	3208549134	0	64	1217	85.4124
HumGenDis_17	92161280	71307493	2404874210	0	48	1358	85.2679
HumGenDis_18	136993488	104609976	3616829974	0	73	1480	90.3568

Supplementary Table 2: List of cancer-related genes studies (n=550). Cancer susceptibility genes (n=105) are shown in **bold.**

ABL1	ABL2	ACVR1	ACVR1B	**AIP**	AKT1	AKT2
AKT3	**ALK**	ALOX12B	ANKRD11	ANKRD26	**APC**	AR
ARAF	ARFRP1	ARID1A	ARID1B	ARID2	ARID5B	ASXL1
ASXL2	**ATM**	ATR	ATRX	AURKA	AURKB	AXIN1
**AXIN2**	AXL	B2M	**BAP1**	BARD1	BBC3	BCL10
BCL2	BCL2L1	BCL2L11	BCL2L2	BCL6	BCOR	BCORL1
BCR	BIRC3	**BLM**	**BMPR1A**	BRAF	**BRCA1**	**BRCA2**
BRD4	**BRIP1**	BTG1	BTK	**BUB1B**	CALR	CARD11
CASP8	CBFB	**CBL**	CCND1	CCND2	CCND3	CCNE1
CD274	CD276	CD74	CD79A	CD79B	**CDC73**	**CDH1**
CDK12	**CDK4**	CDK6	CDK8	CDKN1A	**CDKN1B**	**CDKN1C**
**CDKN2A**	CDKN2B	CDKN2C	**CEBPA**	CENPA	**CEP57**	CHD2
CHD4	CHEK1	**CHEK2**	CHPF2	CIC	CREBBP	CRKL
CRLF2	CSF1R	CSF3R	CSNK1A1	CTCF	CTLA4	CTNNA1
CTNNB1	CUL3	CUX1	CXCR4	**CYLD**	DAXX	DCUN1D1
DDR2	DDX3X	**DDX41**	DHX15	**DICER1**	DIS3	**DIS3L2**
DNAJB1	DNMT1	DNMT3A	DNMT3B	DOT1L	DROSHA	E2F3
EED	EGFL7	**EGFR**	EIF1AX	EIF4A2	EIF4E	EML4
EP300	**EPCAM**	EPHA3	EPHA5	EPHA7	EPHB1	ERBB2
ERBB3	ERBB4	ERCC1	**ERCC2**	**ERCC3**	**ERCC4**	**ERCC5**
ERG	ERRFI1	ESR1	ETS1	ETV1	ETV4	ETV5
**ETV6**	EWSR1	**EZH2**	FAM175A	FAM46C	**FANCA**	**FANCB**
**FANCC**	**FANCD2**	FANCE	FANCF	**FANCG**	FANCI	FANCL
FANCM	**FAS**	FAT1	FBXW7	FGF1	FGF10	FGF14
FGF19	FGF2	FGF23	FGF3	FGF4	FGF5	FGF6
FGF7	FGF8	FGF9	FGFR1	FGFR2	FGFR3	FGFR4
**FH**	**FLCN**	FLI1	FLT1	FLT3	FLT4	FOXA1
FOXL2	FOXO1	FOXP1	FRS2	FTSJ3	FUBP1	FYN
GABRA6	**GATA1**	**GATA2**	GATA3	GATA4	GATA6	GEN1
GID4	GLI1	GNA11	GNA13	GNAQ	GNAS	**GPC3**
GPS2	GREM1	GRIN2A	GRM3	GSK3B	H3F3A	H3F3B
H3F3C	HGF	HIST1H1C	HIST1H2BB	HIST1H2BD	HIST1H3A	HIST1H3B
HIST1H3C	HIST1H3D	HIST1H3E	HIST1H3F	HIST1H3G	HIST1H3H	HIST1H3I
HIST1H3J	HIST2H3A	HIST2H3C	HIST2H3D	HIST3H3	HLA-A	HLA-B
HLA-C	HNF1A	HNRNPK	HOXB13	**HRAS**	HSD3B1	HSP90AA1
ICOSLG	ID3	IDH1	IDH2	IFNGR1	IGF1	IGF1R
IGF2	IKBKE	IKZF1	IL10	IL7R	INHA	INHBA
INPP4A	INPP4B	INSR	IRAK4	IRF2	IRF4	IRS1
IRS2	JAK1	JAK2	JAK3	JUN	KAT6A	KDM5A

KDM5C	KDM6A	KDR	KEAP1	KEL	KIF5B	**KIT**
KLF4	KLHL6	KMT2B	KMT2C	KMT2D	**KRAS**	KRT222
LAMP1	LATS1	LATS2	LMO1	LRP1B	LYN	**LZTR1**
MAD2L2	MAGI2	MALT1	MAP2K1	MAP2K2	MAP2K4	MAP3K1
MAP3K13	MAP3K14	MAP3K4	MAPK1	MAPK3	**MAX**	MBD4
MCL1	MDC1	MDM2	MDM4	MED12	MEF2B	**MEN1**
**MET**	MGA	**MITF**	**MLH1**	MLLT3	MPL	MRE11A
**MSH2**	MSH3	**MSH6**	MST1	MST1R	MTOR	**MUTYH**
MYB	MYC	MYCN	MYD88	MYL6	MYOD1	NAB2
**NBN**	NCOA3	NCOR1	NEGR1	**NF1**	**NF2**	NFE2L2
NFKBIA	NKX2-1	NKX3-1	NOTCH1	NOTCH2	NOTCH3	NOTCH4
NPM1	NRAS	NRG1	**NSD1**	**NTHL1**	NTRK1	NTRK2
NTRK3	NUP93	NUTM1	PAK1	PAK3	**PALB2**	PARK2
PARP1	PAX3	**PAX5**	PAX7	PAX8	PBRM1	PDCD1
PDCD1LG2	**PDGFRA**	PDGFRB	PDK1	PDPK1	PGR	PHF6
**PHOX2B**	PIK3C2B	PIK3C2G	PIK3C3	PIK3CA	PIK3CB	PIK3CD
PIK3CG	PIK3R1	PIK3R2	PIK3R3	PIM1	PLCG2	PLK2
PMAIP1	PMS1	**PMS2**	PNRC1	**POLD1**	**POLE**	**POT1**
PPARG	PPM1D	PPP2R1A	PPP2R2A	PPP6C	PRDM1	PRDM10
PREX2	**PRF1**	**PRKAR1A**	PRKCI	PRKDC	PRSS8	PSMC5
**PTCH1**	PTCH2	**PTEN**	**PTPN11**	PTPRD	PTPRS	PTPRT
QKI	RAB35	RAC1	RAD21	RAD50	RAD51	RAD51B
**RAD51C**	**RAD51D**	RAD52	RAD54L	RAF1	RANBP2	RARA
RASA1	**RB1**	RBM10	**RECQL4**	REL	**RET**	RFWD2
RHEB	RHOA	RICTOR	RIT1	**RNF43**	ROS1	RPS6KA4
RPS6KB1	RPS6KB2	RPTOR	RSPO2	RSPO3	**RUNX1**	RUNX1T1
RYBP	**SBDS**	**SDHA**	**SDHAF2**	**SDHB**	**SDHC**	**SDHD**
SETBP1	SETD2	SF3B1	SH2B3	**SH2D1A**	SHQ1	SLIT2
**SLX4**	SMAD2	SMAD3	**SMAD4**	SMARCA2	**SMARCA4**	**SMARCB1**
SMARCC1	SMARCC2	SMARCD1	SMARCD2	SMARCD3	**SMARCE1**	SMC1A
SMC3	SMO	SNCAIP	SOCS1	SOX10	SOX17	SOX2
SOX9	SPEN	SPOP	SPTA1	SRC	SRSF2	STAG1
STAG2	**STAT3**	STAT4	STAT5A	STAT5B	**STK11**	STK40
**SUFU**	SUZ12	SYK	TAF1	TBX3	TCEB1	TCF3
TCF7L2	**TERT**	TET1	TET2	TFE3	TFRC	**TGFBR1**
TGFBR2	**TMEM127**	TMPRSS2	TNFAIP3	TNFRSF14	TOP1	TOP2A
**TP53**	TP63	TRAF2	TRAF7	**TSC1**	**TSC2**	TSHR
U2AF1	UNK	VEGFA	**VHL**	VTCN1	WISP3	**WT1**
XIAP	XPO1	XRCC2	YAP1	YES1	ZBTB2	ZBTB7A
ZFHX3	ZNF217	ZNF703	ZRSR2			

Supplementary Table 3. The mean age at diagnosis of the most common extra-renal tumours in participants with MPT:RCC+X compared to unselected 100kGP participants with the relevant tumour). Only common extra renal tumours (>10% in participants with RCC+X-MPT) were included. Skin tumours includes melanoma, basal cell carcinoma of skin and squamous cell carcinoma of the skin.

	**Participants with RCC+X-MPT**				**General cancer participants in GEL**			
	**Male (268)**		**Female (173)**		**Male**		**Female**	
	**No.**	**Mean age**	**No.**	**Mean age**	**No.**	**Mean age**	**No.**	**Mean age**
**Breast**	-	-	60	56.4	63	67.5	5094	56.8
**Colorectal**	55	66.2	22	62.8	2123	66.1	1581	64.9
**Hematopoietic**	32	60.2	15	57.3	700	51.1	518	49.4
**Prostate**	80	67.3	-	-	1419	65.4	-	-
**Skin**	76	65.2	34	62.3	1413	63.6	1386	60.4

Supplementary Table 4. Participants (n=9) with multiple primary renal tumours (MPRT) with germline variants with uncertain significance (VUS) in the cancer susceptibility genes with autosomal dominant inheritance(40)**.** CCRCC, clear cell renal cell carcinoma. NOS, not otherwise specified. NA, not available.

Gene	Variants	cDNA	Protein	Participant ID	Tumour (age)
ALK	chr2_29221229_C/T	c.218G>A	p.Trp73Ter	MPRT007	CCRCC (30-35), CCRCC (30-35)
				MPRT008	CCRCC (65-70), CCRCC (65-70)
BMPR1A	chr10:86921569_C/T	c.1216C>T	p.Arg406Cys	MPRT009	RCC NOS (60-65), RCC NOS (NA)
CHEK2	chr22:28725099_A/G	c.470T>C	p.Ile157Thr	MPRT010	CCRCC (45-50), CCRCC (45-50)
MSH6	chr2:47800705_G/A	c.2722G>A	p.Glu908Lys	MPRT011	CCRCC (45-50), CCRCC (NA)
PALB2	chr16:23629953_G/T	c.2201C>A	p.Thr734Asn	MPRT012	Other RCC (65-70), CCRCC (65-70)
POLD1	chr19:50402702_C/T	c.931C>T	p.Arg311Cys	MPRT013	RCC NOS (35-40), RCC NOS (NA)
RUNX1	chr21:34834466_C/T	c.749G>A	p.Arg250His	MPRT014	CCRCC (40-45), CCRCC (40-45)
WT1	chr11:32434939_GGC/-	c.405_407del	p.Pro136del	MPRT015	CCRCC (50-55), CCRCC (NA)

Supplementary Table 5. Participants (n=2) with multiple primary renal tumours (MPRT) with pathogenic or likely pathogenic germline variants in the cancer susceptibility genes with autosomal recessive inheritance. CCRCC, clear cell renal cell carcinoma.

Gene	Variants	cDNA	Protein	Participant ID	Tumour (age)
NBN	chr8:89971213_ATTTGT/A	c.657_661del	p.Lys219Asnfs	MPRT010	CCRCC (45-50), CCRCC (45-50)
RECQL4	chr8:144516069_CCT/C	c.1048_1049del	p.Arg350Glyfs	MPRT003	CCRCC (75-80), other RCC (75-80)

Supplementary Table 6. Participants (n=3) with multiple primary renal tumours (MPRT) with germline variants in the potential cancer susceptibility genes (40)**.** The two truncating variants in MBD4 and NTHL1 genes were pathogenic or likely pathogenic but the missense variant in SBDS was classified as with uncertain significance. CCRCC, clear cell renal cell carcinoma. PRCC, papillary renal cell carcinoma. NOS, not otherwise specified. NA, not available.

**Gene**	**Variants**	**cDNA**	**Protein**	**Participant ID**	**Tumour (age)**
MBD4	chr3:129436705_T/-	c.939del	p.Glu314Lysfs	MPRT004	CCRCC (40-45), CCRCC (NA)
NTHL1	chr16:2046238_G/A	c.268C>T	p.Gln90Ter	MPRT016	PRCC (25-30), PRCC (25-30)
SBDS	chr7:66995330_C/A	c.88G>T	p.Ala30Ser	MPRT017	Other RCC (45-50), other RCC (50-55)

Supplementary Table 7. Participants (n=5) with multiple primary renal tumours (MPRT) with pathogenic or likely pathogenic germline variants in the other autosomal dominantly inherited cancer related genes (40)**.** CCRCC, clear cell renal cell carcinoma. PRCC, papillary renal cell carcinoma. NOS, not otherwise specified. AD, autosomal dominant. AR, autosomal recessive.

Gene	Associated inherited disease	Variants	cDNA	Protein	Participant ID	Tumour (age)
ASXL1	Bohring-Opitz syndrome	fo3	c.1934dup	p.Gly646Trpfs	MPRT018	Other RCC (75-80), other RCC (75-80)
KMT2B	Early onset dystonia	chr19:35721219_C/CCC	c.1873_1874dup	p.Pro626Leufs	MPRT019	CCRCC (60-65), PRCC (60-65)
KMT2C	Kleefstra syndrome	chr7:152177063_T/-	c.8390del	p.Lys2797fs	MPRT020	RCC NOS (60-65), RCC NOS (65-70)
SH2B3	Thrombocythemia	chr12:111446790_T/C	c.770T>C	p.Ile257Thr	MPRT017	Other RCC (45-50), other RCC (50-55)
SUZ12	Imagawa-Matsumoto syndrome	chr17:31998954_CAGAT/C	c.2172_2175del	p.Asp725Valfs	MPRT021	RCC NOS (65-70), RCC NOS (65-70)

Supplementary Table 8. Participants (n=9) with a renal cell carcinoma (RCC) and an extra renal tumours who had germline variants with uncertain significance (VUS) in the renal cell carcinoma (RCC) associated cancer susceptibility genes (40)**.**

Gene	Tumour susceptibility	Variant	cDNA	Protein	Participant ID	Tumour (age)
BAP1	Uveal/cutaneous melanoma, mesothelioma, rhabdoid meningiomas, basal cell carcinoma, pancreatic cancer (AD)	chr3_52407978_T/G	c.355A>C	p.Thr119Pro	MPT28	prostate (55-60), RCC (55-60)
CHEK2	Breast cancer, colorectal cancer, prostate cancer, pancreatic cancer, ovarian cancer, thyroid cancer, (AD)	chr22_28694066_G/A	c.1556C>T	p.Thr519Met	MPT29	colorectal (40-45), RCC (60-65)
					MPT30	skin (75-80), RCC (80-85)
		chr22_28696957_C/T	c.1039G>A	p.Asp347Asn	MPT06	breast (50-55), colorectal (60-65) ovary (50-55), RCC (60-65)
		chr22_28711950_T/A	c.880A>T	p.Ile294Phe	MPT31	bladder (85-90), skin (melanoma), (80-85), RCC (80-85)
**FH**	Leiomyomas, rarely leiomyosarcoma, pheochromocytoma, paraganglioma (AD, AR)	chr1_241497927_A/ATTT	c.1433_1434insAAA	p.Lys477dup	MPT32	prostate (40-45), RCC (40-45)
TSC2	Angiomyolipoma, subependymal giant cell astrocytoma,	chr16_2053409_G/A	c.293G>A	p.Arg98Gln	MPT33	skin (70-75), prostate (80-85), RCC (80-85)

	rhabdomyoma (AD)	chr16_2064298_C/G	c.1470C>G	p.Ile490Met	MPT20	skin (70-75), RCC (75-80)
		chr16_2079356_C/T	c.3212C>T	p.Thr1071Ile	MPT34	colorectal (60-65), RCC (70-75)

Supplementary Table 9. Participants (n=35) with a renal cell carcinoma (RCC) and an extra renal tumours that had germline variants with uncertain significance (VUS) in the other cancer susceptibility genes with autosomal dominant inheritance (40).

Gene	Tumour susceptibility	Variant	cDNA	Protein	Participant ID	Tumour (age)
ALK	Neuroblastoma (AD)	chr2_29222587_A/G	c.3380T>C	p.Phe1127Ser	MPT35	ovary (70-75), RCC (70-75)
APC	Colorectal cancer, hepatoblastoma, desmoid tumour (AD)	chr5_112837825_C/T	c.2231C>T	p.Ser744Phe	MPT36	colorectal (55-60), skin (40-45), RCC (55-60)
		chr5_112840810_A/G	c.5216A>G	p.Lys1739Arg	MPT37	skin (55-60), RCC (80-85)
		chr5_112842288_C/G	c.6694C>G	p.His2232Asp	MPT38	prostate (65-70), RCC (65-70)
		chr5_112843351_G/T	c.7757G>T	p.Ser2586Ile	MPT39	skin (70-75), RCC (70-75)
ATM	Breast cancer (AD) and lymphoid haematological malignancy (AR)	chr11_108330381_T/G	c.7475T>G	p.Leu2492Arg	MPT40	breast (70-75), RCC (70-75)
AXIN2	Oligodontia-colorectal cancer syndrome, AD)	chr17_65558146_C/G	c.475G>C	p.Asp159His	MPT41	lung (70-75), RCC (70-75)
BRCA2	Breast cancer, ovarian cancer, prostate cancer, pancreas cancer (AD), and myeloid haematological	chr13_32370991_T/G	c.8523T>G	p.Phe2841Leu	MPT42	uterus (35-40), RCC (30-35)

	malignancy (AR).					
BRIP1	Breast cancer and ovarian cancer (AD), and myeloid haematological malignancy, squamous cell carcinoma (AR)	chr17_61776517_A/G	c.1981T>C	p.Cys661Arg	MPT43	prostate (65-70), RCC (70-75)
		chr17_61808544_G/T	c.841C>A	p.His281Asn	MPT44	colorectal (80-85), RCC (80-86)
		chr17_61849223_A/G	c.413T>C	p.Leu138Ser	MPT12	thrombocythemia (60-65), RCC (60-65)
CBL	Juvenile myelomonocytic leukaemia (AD)	chr11_119285036_T/C	c.1499T>C	p.Leu500Pro	MPT45	colorectal (80-85), prostate (80-85), skin (75-80), RCC (80-85)
CEBPA	Myeloid haematological malignancy (AD)	chr19_33301379_G/A	c.1036C>T	p.Pro346Ser	MPT46	skin (50-55), RCC 55-60)
DICER1	Pleuropulmonary blastoma, cystic nephroma, and ovarian sex cord tumour, Wilms tumour (AD)	chr14_95091086_G/A	c.5551C>T	p.Arg1851Cys	MPT47	prostate (75-80), RCC (75-80)
EGFR	Non-small cell lung cancer (AD)	chr7_55173087_G/A	c.2024G>A	p.Arg675Gln	MPT48	prostate (75-80), RCC (75-80)
KIT	Gastrointestinal stromal tumour (AD)	chr4_54729451_T/C	c.2107T>C	p.Tyr703His	MPT49	skin (75-80), RCC (75-80)
		chr4_54738493_G/A	c.2867G>A	p.Arg956Gln	MPT50	skin (70-75), RCC (80-85)

MEN 1	Parathyroid pituitary adenoma, neuroendocrine tumour, carcinoid tumour, adrenocortical carcinoma (AD)	chr11_64806319_T/C	c.962A>G	p.Tyr321Cys	MPT51	uterus (50-55), RCC (60-65)
MSH 6	Colorectal cancer, endometrial cancer and ovarian cancer (AD), brain tumour, haematological malignancy and embryonal tumour (AR).	chr2_47799650_A/G	c.1667A>G	p.Tyr556Cys	MPT13	thyroid (60-65), RCC (55-60)
PAL B2	Breast cancer and pancreas cancer (AD), myeloid haematological malignancy, medulloblastoma, neuroblastoma, and Wilms tumours (AR)	chr16_23626378_G/C	c.2606C>G	p.Ser869Cys	MPT52	uterus (70-75), RCC (70-75)
PMS 2	Colorectal cancer, endometrial cancer and ovarian cancer (AD), brain tumour, haematological malignancy and supratentorial primitive	chr7_6002515_C/T	c.475G>A	p.Val159Met	MPT53	breast (NOS), thyroid (NOS), uterus (NOS), RCC (NOS)

	neuroectodermal tumour (AR)					
POLD1	Colorectal cancer and endometrial cancer (AD)	chr19_50402066_C/CG	c.532_532insG	p.Arg180Glufs	MPT54	skin (70-75), RCC (70-75)
POLE	Colorectal cancer (AD, AR)	chr12_132668866_T/C	c.1868A>G	p.Tyr623Cys	MPT15	colorectal (70-75), RCC (65-70)
					MPT55	colorectal (75-80), RCC (70-75)
		chr12_132675756_T/C	c.1085A>G	p.Tyr362Cys	MPT56	cervix (40-45), RCC (65-70)
		chr12_132680674_T/C	c.218A>G	p.Asp73Gly	MPT57	adrenal (40-45), RCC (30-35)
RAD51D	Breast-ovarian cancer (AD)	chr17_35100985_G/A	c.955C>T	p.Gln319Ter	MPT58	colorectal (50-55), RCC (45-50)
		chr17_35103492_G/A	c.689C>T	p.Ala230Val	MPT59	breast (60-65), colorectal (70-75), lymphoma (55-60), RCC (65-70)
RUNX1	Myeloid haematological malignancy (AD)	chr21_34887039_A/T	c.155T>A	p.Met52Lys	MPT16	skin (50-55), RCC (60-65)
SMARCB1	Rhabdoid tumour, central primitive neuroectodermal tumour (AD)	chr22_23816839_T/A	c.698T>A	p.Phe233Tyr	MPT60	lung (55-60), thyroid (55-60), pituitary (70-75),

						RCC (30-35)
STK11	Colorectal cancer, gastric cancer, breast cancer, sex cord-stromal cancer, pancreatic cancer, osteosarcoma, (AD)	chr19_1223095_T/C	c.1031T>C	p.Leu344Pro	MPT07	acute lymphoblastic leukaemia (0-5), RCC (25-30)
SUFU	Medulloblastoma, meningioma (AD)	chr10_102615273_G/A	c.1028G>A	p.Arg343His	MPT61	colorectal (65-70), skin (80-85), RCC (80-85)
TERT	Acute myeloid leukaemia, squamous cell carcinoma, melanoma (AD)	chr5_1293376_A/G	c.1510T>C	p.Ser504Pro	MPT62	skin (squamous cell carcinoma) (55-60), RCC (60-65)
TMEM127	Pheochromocytoma (AD)	chr2_96253927_G/T	c.598C>A	p.Pro200Thr	MPT63	breast (60-65), schwannoma (55-60), skin (65-70), RCC (60-65)
**TP53**	Breast cancer, sarcoma, lung cancer, adrenocortical carcinoma, glioblastoma, astrocytoma (AD)	chr17_7675070_C/T	c.542G>A	p.Arg181His	MPT64	thyroid (50-55), polycythaemia (50-55), RCC (50-55)

Supplementary Table 10. The cancer records of the 5 participants with a renal cell carcinoma (RCC) and an extra renal tumours that were identified as carriers of pathogenic or likely pathogenic variants in the other cancer susceptibility genes with autosomal recessive inheritance.

Gene	Tumour susceptibility	Variant	cDNA	Protein	Participant ID	Tumour (age)
BLM	Haematological malignancy, squamous cell carcinoma, colorectal cancer, breast cancer	chr15_90784953_C/T	c.2695C>T	p.Arg899Ter	MPT65	thyroid (70-75), RCC (70-75)
					MPT66	breast (60-65), RCC (65-70)
ERCC3	Basal cell carcinoma, squamous cell carcinoma, melanoma, breast cancer, lung cancer	chr2_127292785_A/G	c.296T>C	p.Phe99Ser	MPT67	lung (75-80), RCC (75-80)
ERCC4	Basal cell carcinoma, squamous cell carcinoma, melanoma, lung cancer	chr16_13935413_TAACTC/T	c.1482_1486del	p.Thr495Asnfs	MPT68	lymphoma (45-50), RCC (60-65)
FANCA	Myeloid haematological malignancy, squamous cell carcinoma,	chr16_89765062_T/G	c.2606A>C	p.Gln869Pro	MPT69	breast (70-75), RCC (70-75)

Supplementary Table 11. Participants (n=11) with a renal cell carcinoma (RCC) and an extra renal tumours that had pathogenic or likely pathogenic germline variants in the other cancer related genes (40)**.** AD, autosomal dominant. AR, autosomal recessive.

Gene	Associated disease	Variant	cDNA	protein	Participant ID	Tumour (age)
DNMT3A	Heyn-Sproul-Jackson syndrome, Tatton-Brown-Rahman syndrome (AD)	chr2_25240366_C/T	c.2258G>A	p.Trp753Ter	MPT70	Gastric (70-75), head/neck (60-65), prostate (65-70), RCC(60-65), skin (65-70)
		chr2_25236968_G/A	c.2446C>T	p.Gln816Ter	MPT71	RCC (70-75), skin (70-75)
		chr2_25234374_G/A	c.2644C>T	p.Arg882Cys	MPT72	Lung (70-75), RCC(65-70)
FANCF	Fanconi anemia (AR)	chr11_22625233_C/CT	c.577_578insA	p.Trp193Ter	MPT73	RCC (75-80), uterus (75-80)
FANCI	Fanconi anemia (AR)	chr15_89303898_G/A	c.3041G>A	p.Cys1014Tyr	MPT74	Bladder (85-90), RCC (75-80), skin (55-60)
FGF5	Trichomegaly (AR)	chr4_80266832_T/A	c.8T>A	p.Leu3Ter	MPT75	Prostate (70-75), RCC (75-80)
KMT2B	Dystonia, childhood-onset (AD)	chr19_35720592_A/AC	c.1245_1246insC	p.Leu417Profs	MPT76	Prostate (60-65), RCC (75-80)
		chr19_35721215_C/CCA	c.1868_1869insCA	p.Ala624Lysfs	MPT77	Prostate (65-70), RCC (75-80)

PDGFRB	Basal ganglia calcification, idiopathic, Kosaki overgrowth syndrome, Myeloproliferative disorder with eosinophilia (AD)	chr5_150123142_G/A	c.2083C>T	p.Arg695Cys	MPT78	Colorectal (50-55), RCC (65-70)
RAD50	Nijmegen breakage syndrome-like disorder (AR)	chr5_132587637_C/T	c.832C>T	p.Arg278Ter	MPT79	Colorectal (55-60), RCC (55-60)
SH2B3	Thrombocythemia (AD)	chr12_111447491_G/A	c.1183G>A	p.Glu395Lys	MPT80	RCC (65-70), sarcoma (65-70)

Supplementary Table 12. The area under the curve (AUC) result to compare the different age threshold for the yield of pathogenic or likely pathogenic variants in cancer susceptibility genes in participants with multiple primary renal tumours (MPRT) and participants with a renal cell carcinoma and an extra renal tumour (MPT:RCC+X).

Age of RCC		≤35	≤40	≤45	≤50	≤55	≤60	≤65
**MPRT: first RCC**	**Male**	0.60	0.69	**0.77**	0.68	0.64	0.57	0.52
	**Female**	0.67	0.60	0.58	0.54	0.68	**0.76**	0.72
	**All**	0.63	0.65	**0.69**	0.61	0.64	0.64	0.59
**MPRT: second RCC**	**Male**	0.49	**0.72**	0.70	0.60	0.55	0.51	0.59
	**Female**	0.67	0.67	0.67	0.61	0.61	0.70	**0.82**
	**All**	0.59	**0.68**	0.66	0.58	0.54	0.57	0.60
**All MPT:RCC+X**	**Male**	0.54	0.57	0.60	**0.68**	0.55	0.58	0.61
	**Female**	0.48	0.51	0.55	0.51	0.52	0.55	**0.68**
	**All**	0.51	0.54	0.58	0.55	0.54	0.57	**0.66**
**MPT:RCC+X excluding lung cancer, non-melanoma and cervical cancer**	**Male**	0.54	0.58	**0.61**	0.59	0.55	0.59	0.58
	**Female**	0.48	0.50	0.55	0.51	0.52	0.55	**0.68**
	**All**	0.51	0.54	0.58	0.55	0.54	0.58	**0.65**
